# IDHwt glioblastomas can be stratified by their transcriptional response to standard treatment, with implications for targeted therapy

**DOI:** 10.1186/s13059-024-03172-3

**Published:** 2024-02-07

**Authors:** Georgette Tanner, Rhiannon Barrow, Shoaib Ajaib, Muna Al-Jabri, Nazia Ahmed, Steven Pollock, Martina Finetti, Nora Rippaus, Alexander F. Bruns, Khaja Syed, James A. Poulter, Laura Matthews, Thomas Hughes, Erica Wilson, Colin Johnson, Frederick S. Varn, Anke Brüning-Richardson, Catherine Hogg, Alastair Droop, Arief Gusnanto, Matthew A. Care, Luisa Cutillo, David R. Westhead, Susan C. Short, Michael D. Jenkinson, Andrew Brodbelt, Aruna Chakrabarty, Azzam Ismail, Roel G. W. Verhaak, Lucy F. Stead

**Affiliations:** 1 Leeds Institute of Medical Research at St James’s, University of Leeds, Leeds, UK; 2 Leeds Institute of Cardiovascular and Metabolic Medicine, University of Leeds, Leeds, UK; 3 The Walton Centre NHS Foundation Trust, Liverpool, UK; 4 School of Science, Technology and Health, York St John University, York, YO31 7EX UK; 5 The Jackson Laboratory for Genomic Medicine, Farmington, CT USA; 6 School of Applied Sciences, University of Huddersfield, Huddersfield, UK; 7 Welcome Sanger Institute, Hinxton, Saffron Walden, UK; 8 School of Mathematics, University of Leeds, Leeds, UK; 9 School of Molecular and Cellular Biology, University of Leeds, Leeds, UK; 10 Leeds Teaching Hospital, Leeds, UK; 11 Institute of Systems, Molecular and Integrative Biology, University of Liverpool, Liverpool, UK; 12 Yale School of Medicine, New Haven, CT USA

## Abstract

**Background:**

Glioblastoma (GBM) brain tumors lacking *IDH1* mutations (IDHwt) have the worst prognosis of all brain neoplasms. Patients receive surgery and chemoradiotherapy but tumors almost always fatally recur.

**Results:**

Using RNA sequencing data from 107 pairs of pre- and post-standard treatment locally recurrent IDHwt GBM tumors, we identify two responder subtypes based on longitudinal changes in gene expression. In two thirds of patients, a specific subset of genes is upregulated from primary to recurrence (Up responders), and in one third, the same genes are downregulated (Down responders), specifically in neoplastic cells. Characterization of the responder subtypes indicates subtype-specific adaptive treatment resistance mechanisms that are associated with distinct changes in the tumor microenvironment. In Up responders, recurrent tumors are enriched in quiescent proneural GBM stem cells and differentiated neoplastic cells, with increased interaction with the surrounding normal brain and neurotransmitter signaling, whereas Down responders commonly undergo mesenchymal transition. ChIP-sequencing data from longitudinal GBM tumors suggests that the observed transcriptional reprogramming could be driven by Polycomb-based chromatin remodeling rather than DNA methylation.

**Conclusions:**

We show that the responder subtype is cancer-cell intrinsic, recapitulated in in vitro GBM cell models, and influenced by the presence of the tumor microenvironment. Stratifying GBM tumors by responder subtype may lead to more effective treatment.

**Supplementary Information:**

The online version contains supplementary material available at 10.1186/s13059-024-03172-3.

## Background

Glioblastoma (GBM) brain tumors are currently incurable. GBM cancer cells infiltrate the surrounding normal brain so, although patients undergo surgery, complete resection is not possible. Standard of care includes subsequent chemoradiation with temozolomide (TMZ), but this only modestly prolongs survival: typically tumors recur 6–9 months later and are almost always fatal. In order to more effectively treat GBM, we must understand why some unresected GBM cells survive chemoradiation. To this end, global efforts have characterized clonal evolution through the treatment of GBM. We performed the largest of such genomic studies and, in agreement with Körber et al., concluded that treatment resistance is not driven by somatic mutations in specific genes or pathways [1, 2]. Single-cell analyses of primary GBM tumors have identified transcriptionally defined neoplastic cell states that are shared across genomic subclones and patients, and exhibit high levels of plasticity [3–5]. Single-cell epigenetic profiling of primary tumors concurs with this notion, having linked chromatin accessibility with the observed neoplastic transcriptional states [6, 7]. Together, these findings suggest that epigenetics underpin GBM cell behaviors, including those that enable inherent or adaptive treatment resistance.

Our subsequent efforts have focused on characterizing the changes in transcriptional profiles through treatment. Our initial investigation of bulk longitudinal glioma profiles indicated a convergence upon specific phenotypes at recurrence [8]. However, distinctly different trajectories occur for tumors that are wild-type for isocitrate dehydrogenases (IDHwt) compared to those harboring mutations in the genes encoding these enzymes (IDHmut) [8], indicating that these two entities require separate analysis [8]. Wang et al. [9] previously showed that primary GBM cancer cells reside on a single axis of variation between proneural and mesenchymal phenotypes [5]. This group subsequently performed multi-omic, single-cell analyses of longitudinal IDHwt GBM tumors and showed that this is also true for recurrent tumors but that treatment alters the prevalence of cells along this axis, whilst highlighting mechanisms by which a mesenchymal shift may occur [9].

The study described herein expands upon our previous bulk tumor studies by performing transcriptional analysis of a larger cohort (*n* = 214) of paired longitudinal GBMs that are specifically IDHwt and recurred locally following standard treatment. We identify a subset of genes, linked by an epigenetic remodeling complex, that are consistently dysregulated through standard of care but in opposite directions in different patients. This defines two responder subtypes: “Up” and “Down” responders. We show that this phenomenon is cancer cell-intrinsic but affected by the presence of normal brain infiltration. Responder subtype stratification of IDHwt GBM patients is biologically and clinically meaningful, suggesting distinct treatment resistance mechanisms and therapeutic vulnerabilities. To investigate this further, we identified in vitro models that recapitulate the responder subtypes, albeit with effect sizes much smaller than we observe in patients. We went on to show that models that incorporate the TME may be required for downstream mechanistic work and drug testing. Our work confirms our previous findings in longitudinal bulk samples and those of Wang et al. at single-cell resolution, whilst providing additional context and a metric for testing which model systems can be used to test personalized medicine approaches and maximize translational impact [8, 9].

## Results

### Overview of Discovery and Validation cohorts

**Fig. 1:**
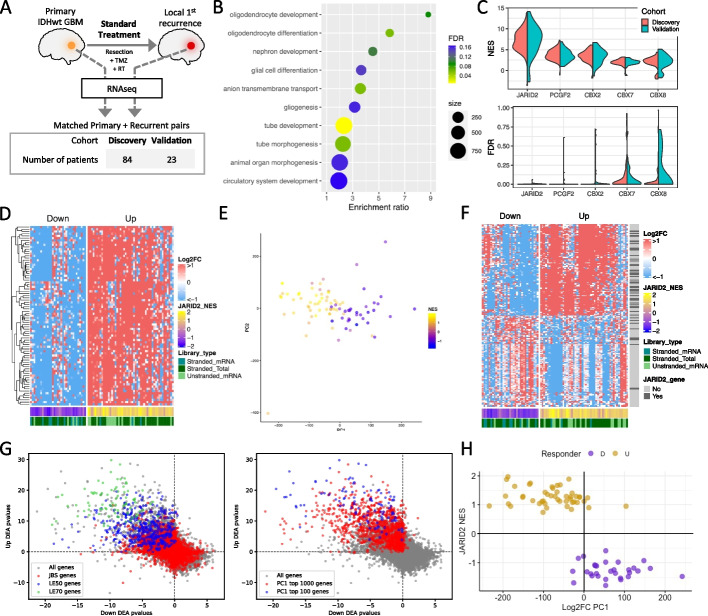
**A** Schematic of the study design and cohort sizes. These panels visualize data from the Discovery cohort (Validation data is in supplemental figures). **B** Biological processes enriched in the genes differentially expressed between matched primary and recurrent GBMs. **C** Per-patient normalized enrichment scores (NES, top plot) and false discovery rates (FDR, bottom plot) for top-scoring promoter-binding factors associated with longitudinal gene expression changes. **D** Heatmap of the longitudinal fold change in expression for each patient (columns) for the JARID2 binding sites genes (JBSgenes) in the leading edge of > 50% of patients (LE50 genes, rows). Patients separate into Up (NES > 0) and Down (NES < 0) responders irrespective of RNAseq library preparation approach. **E** Patients are plotted, colored by JBSgenes NES, according to principal components 1 (PC1) and 2 (PC2) of their whole transcriptome longitudinal fold change in expression. **F** Heatmap of the longitudinal fold change in expression for each patient (columns) for the largest 100 positive and largest 100 negative weighted genes of PC1 from panel **E** (rows). Whether each gene is a JBSgene is also indicated. **G** Each gene is plotted according to its -log_10_p-value result of separate differential expression analyses (DEA) in matched recurrent vs primary tumors in Down (*x*-axis) and Up (*y*-axis) responders. Left plot: genes colored according to whether they are JBSgenes or, more specifically, LE50 and LE70 genes. Right plot: genes colored according to whether they are in the top 100 or 1000 genes ranked by the absolute value of PC1 from the analysis in panel **E**. **H** Plotting patients according to their JBSgene NES and PC1 score from panel **E** clearly separates Up and Down responders

We restricted our study to de novo IDHwt GBM tumors and matched, post-standard treatment (having received radiation and Temozolomide), locally recurrent tumors. The Discovery cohort consisted of 168 longitudinally paired samples from 84 patients with RNAseq data processed locally. The Validation cohort consisted of 46 paired samples from 23 patients for which RNAseq data was processed via a distinct pipeline within the Glioma Longitudinal AnalySiS (GLASS) consortium [8] (Fig. 1A; Additional file 1: Table S1). Although GLASS has a considerable number of paired samples, these were the only ones distinct from the Discovery cohort and with sufficient metadata to fit the inclusion criteria. Sequencing metrics for the Discovery cohort are given in Additional file 1: Table S2.

### Longitudinal changes in gene expression delineate two responder subtypes in GBM

The genes significantly differentially expressed between matched primary and recurrent GBMs were enriched for terms associated with neurodevelopment (Fig. 1B). Neurodevelopment is orchestrated by master regulators of gene expression, where cascades of transcription factors work in concert with chromatin remodeling complexes. To investigate whether specific regulators were implicated in the genes dysregulated in primary versus recurrent GBMs, we created novel, comprehensive gene sets for DNA-binding factors. We did this by acquiring publicly available data for 785 DNA binding factors that had undergone ChIPseq and assigning a gene to a factor’s set if its promoter harbored a binding peak for that factor in at least two independent experiments (see the “ Methods” section). Subsequent gene set enrichment analysis showed that genes containing a JARID2 (Jumonji and AT-Rich Interacting Domain 2) binding site in their promoter (JBSgenes) were consistently and significantly altered through treatment across patients in both the Discovery and Validation cohorts (Fig. 1C; Additional file 1: Tables S3-12). This result was consistent when the definition of a promoter was extended from 1 to 2 kb or 5 kb on either side of the transcription start site (TSS) and when quantifying expression at the level of individual TSSs, rather than genes (Additional file 2: Figs. S1A and B; Additional file 1: Tables S13-18).

To assess whether the same JBSgenes were driving the enrichment across patients, we inspected the stability of inclusion within the leading edge. In a total of 5234 JBSgenes, 443 were LE50 (genes in the leading edge of at least 50% of patients) and 81 were LE70 in the Discovery cohort. These significantly overlapped with the LE50 (444 genes) and LE70 (87 genes) calculated independently in the Validation cohort (hypergeometric test *P* = 8.2E − 217 and *P* = 6.2E − 40 and representation factors of 7.3 and 24.5 respectively) (Additional file 1: Table S19). The fold change in expression from primary to recurrence (Log2FC) for LE70 genes was in a consistent direction within, but differed between, patients (Fig. 1D). In 60% of patients, LE70 genes were upregulated from primary to recurrence (coined Up responders), and in the remaining 40%, the same genes were downregulated (Down responders). This phenomenon was recapitulated in the Validation cohort with the same split of Up and Down responders (chi-squared test *P* = 0.98; Additional file 2: Fig. S1C).

JARID2 is an accessory protein to Polycomb Repressive Complex 2 (PRC2), which is responsible for gene repression via deposition of H3K27me3. PRC2 has a role in the transcriptional reprogramming required for neurodevelopment and brain cell lineage determination. To investigate transcriptional reprogramming from primary to recurrent GBM, we performed principal component analysis on Log2FC profiles. Principal component 1 (PC1), the main source of variation, separated patients according to the strength of their classification as Up or Down responders, quantified by their JARID2 gene set normalized enrichment score (NES), in both the Discovery (Fig. 1E, Additional file 1: Table S20) and Validation cohort (Additional file 2: Fig. S1D, Additional file 1: Table S21). The 100 genes with the highest PC1 loadings were significantly enriched for JBSgenes (chi-squared test *P* = 1.8E − 5; Fig. 1F). This indicates that Up and Down responder tumors undergo transcriptional reprogramming in opposing directions through treatment, driven by a key set of genes but propagated throughout the transcriptome. Splitting patients by responder subtype and performing separate primary versus recurrent differential gene expression analyses further confirmed that the same genes are being dysregulated in different directions, with the most significant being enriched in JBSgenes and those with the highest PC1 loadings (Fig. 1G; Additional file 1: Table S22-23). Plotting patients according to their Log2FC PC1 value and their JARID2 NES resulted in a clear separation of Up and Down responders (Fig. 1H).

### Responder subtypes undergo different changes in neoplastic, normal brain, and immune cell populations through treatment

**Fig. 2:**
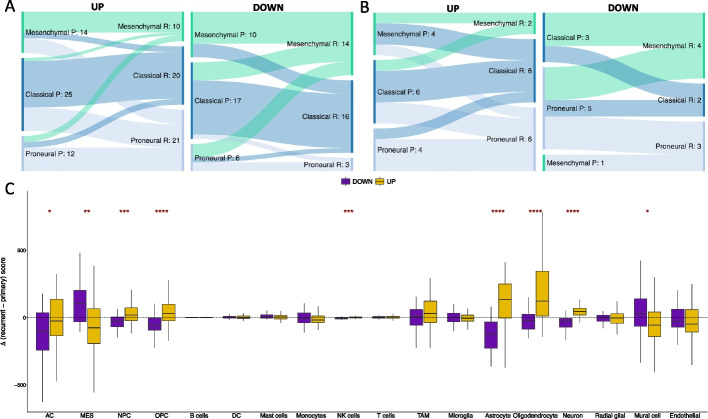
**A** Sankey plots showing the prevalence of subtype switching from primary (P) to recurrent (R) GBM in the Up responders (left) and Down responders (right) in the Discovery cohort. **B** The same as panel A but for the Validation cohort. **C** The distributions of change in cell type score, assigned per sample by GBMdeconvoluteR, between primary and matched recurrent GBMs in Down (purple) and Up (gold) responders. The horizonal dotted line indicates no change. The median is denoted by a black horizontal line. Significance is denoted by asterisks: *p < 0.05; **p < 0.01; ***p < 0.001; ****p < 0.0001. Neoplastic GBM cells are on the left of the plot: AC, astrocyte-like; MES, mesenchymal-like; NPC, neural progenitor-like; OPC, oligodendrocyte progenitor-like

We proceeded to investigate the clinical and biological differences between responder subtypes. In multivariate survival analysis, including known prognostic GBM markers (i.e., expression of O-6-Methylguanine-DNA Methyltransferase (*MGMT)* in the primary tumor and age at diagnosis), there was no association between responder type and progression-free (*P* = 0.59, *β*=  − 0.86) or overall (*P* = 0.97, *β* = 0.1) survival. The prevalence of classical, mesenchymal, and proneural GBM subtypes in primary tumors is the same for both responder subtypes (chi-squared *P* = 0.98), and the probability of switching subtype between primary and recurrence remains the same (chi-squared *P* = 0.89). However, there is a significant difference in subtype representation between responder subtypes at recurrence (chi-squared *P* = 0.0030 Fig. 2A). In Up responders, the majority of samples that switch subtype become proneural (60%, *n* = 16) whereas in Down responders there is a significant switch to mesenchymal subtype (61%, *n* = 11). The same is observed in the Validation cohort where 43% of Up responders that switch become proneural (*n* = 7) and 67% of Down responders that switch become mesenchymal (*n* = 6) (Fig. 2B).

GBM tumor subtypes, defined by bulk RNAseq profile clustering, result from the dominant single GBM cell type signal in the tumor at any given timepoint [3]. The prevalence of single GBM cell types is influenced by the tumor microenvironment [3]. To investigate whether the opposing gene dysregulation observed in Up and Down responders resulted from different cell population dynamics, we applied GBMdeconvoluteR [10], a GBM-specific neoplastic, normal brain, and immune cell deconvolution tool, to our datasets (Fig. 2C and Additional file 2: Fig. S2A). GBMdeconvoluteR uses the neoplastic GBM cell classifications derived in Neftel et al., quantifying the prevalence of mesenchymal (MES), astrocyte cell-like (AC), neural progenitor cell-like (NPC), and oligodendrocyte progenitor cell-like (OPC) cancer cells [3]. We found that longitudinal neoplastic cell population changes are significantly different between the responder subtypes (Fig. 2C): Down responders predominantly increase in MES GBM cells (Wilcoxon *P* = 1.6E − 3) and decrease in both NPC (Wilcoxon *P* = 8.0E − 4) and OPC (Wilcoxon *P* = 5.6E − 5) cells. In Up responder, the opposite is true, with OPC cells increasing the most from primary to recurrent. In both responder subtypes AC cells are more likely to decrease post-treatment, but significantly more so in Down responders than Up (Wilcoxon *P* = 0.047). These subtype-specific changes in neoplastic cells are also observed in the Validation dataset (Additional file 2: Fig. S2A). We also found significant differences between the responder subtypes with regard to how normal brain cell populations change from primary to recurrence (Fig. 2C). Astrocytes, oligodendrocytes, and neurons were all found to be significantly increased in the Up responders, post-treatment, but decreased in Down responders (Wilcoxon *P* = 1.6E − 7, *P* = 6.6E − 6 and *P* = 1.1E − 10 respectively), as confirmed in the Validation cohort (Additional file 2: Fig. S2A).

Conversely, changes in immune cell population are less evident through treatment in Up and Down responders, and significant findings differ between the Discovery cohort (where NK cell infiltration is significantly increased in Up responders: Wilcoxon *P* = 9.8E − 4 Fig. 2C) and Validation cohort (where T cell infiltration is significantly increased in Up responders: Wilcoxon *P* = 0.016, and monocyte infiltration is significantly increased in Down responders Wilcoxon *P* = 0.028; Additional file 2: Fig. S2A). Upon re-classifying cells as lymphoid or myeloid across both datasets, we found that Up responders show a significant increase in lymphoid cells, post-treatment, compared to Down responders (Wilcoxon *P* = 8.8E − 4 Additional file 2: Fig. S2B).

These findings raise the possibility that responder classification is driven by tumor purity, rather than being an intrinsic neoplastic cell phenomenon, so we proceeded to investigate this further using single-cell data [9].

### GBM responder subtyping is driven by neoplastic cells but affected by tumor purity

**Fig. 3:**
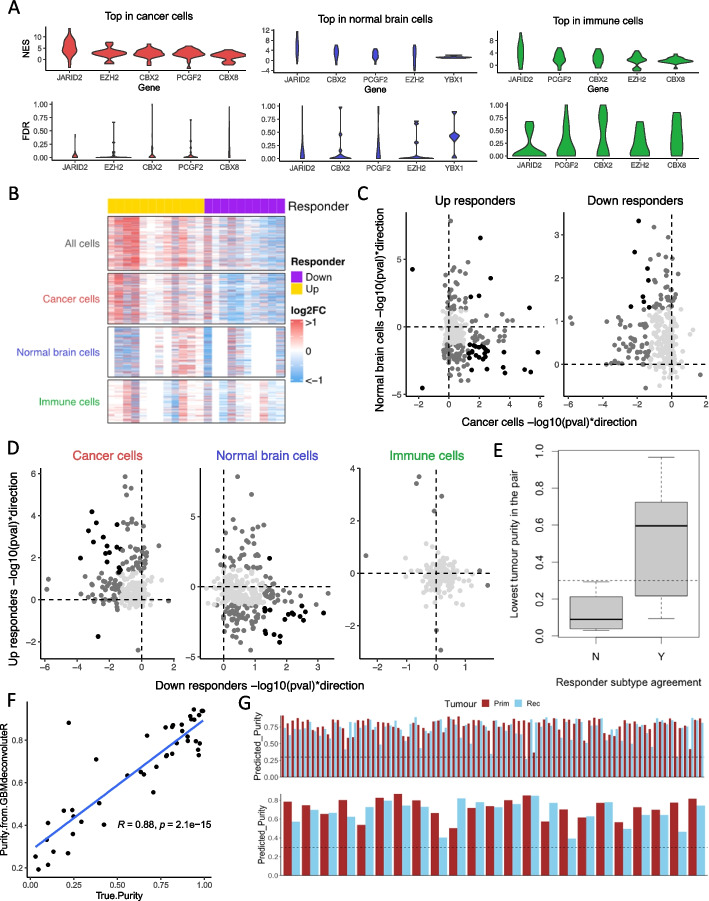
**A** Per-patient normalized enrichment scores (NES, top plot) and false discovery rates (FDR, bottom plot) for the top-scoring promoter-binding factors associated with longitudinal gene expression changes in purified pseudo-bulk samples formed by combining single-cell profiles for: cancer cells (left), normal brain cells (middle), and immune cells (right). **B** Heatmaps showing the longitudinal fold-change in expression (Log2FC) of LE50 genes (rows) in pseudo-bulk samples consisting of all cells, or purified cell subsets. The responder subtype for each patient (columns), derived from the “all cell” pseudo-bulk, is indicated by the top color bar. **C**, **D** LE50 genes plotted according to their direction and significance of dysregulation through treatment in cancer cells (*x*-axis) and normal brain cells (*y*-axis) when differential expression analysis is performed separately in Up (left) and Down (right) responders (**C**); or in Down responders (*x*-axis) versus Up responders (*y*-axis) when differential expression analysis is performed separately in pseudo-bulk samples of pure cancer cells (left), normal brain cells (middle), or immune cells (right) (**D**). Light gray: *p* > 0.05 in both comparison; dark gray: *p* < 0.05 in one; black: *p* < 0.05 in both. **E** Boxplots of lowest tumor purity (cancer cells as a proportion of all cells) in a longitudinal pair split by to whether that patient’s responder subtype agreed between the cancer cell subset and full tumor pseudo-bulk (Y) or not (N). Dotted line = 30% tumor purity. **F** True purity of each GBM sample plotted against the purity predicted by applying GBMdeconvoluteR and using the resulting scores in the formula (MES + AC)/(MES + AC + B + DC + Mast + NK + T + Oligodendrocytes). Shaded area: 95% confidence interval. **G** Predicted tumor purity for Discovery cohort (top) and the Validation cohort (bottom) samples. Dotted line: 30% tumor purity

We acquired single-cell expression data for 22 paired IDHwt GBMs from the Gene Expression Omnibus (accession code GSE174554) [9]. For each sample, cells were separated into three fractions: cancer cells, immune cells, and normal brain cells. All 44 samples had at least 50 cancer cells but only 68% (*n* = 30) had sufficient normal brain cells, and only 77% (*n* = 34) had sufficient immune cells. Pseudo-bulk expression profiles were created for each purified cell population per patient and underwent gene set enrichment analysis using our novel DNA-binding factor gene sets. JBSgenes were the most enriched in all cell subsets, but most significantly in the cancer cell subset (Fig. 3A). The list of LE50 genes derived separately from each cell subset was compared with that from the Discovery cohort; there was a significantly greater overlap for the cancer cell fraction compared to both the immune (chi-squared *P* = 0.013) and normal brain cell fraction (chi-squared *P* = 0.046). This suggests that the cancer cell fraction is driving the phenomenon we observe in the larger cohorts of patient samples. We created additional pseudo-bulk profiles from all the cells of each tumor. The responder subtype calls from the full tumor (which resulted in 12 Up and 10 Down responders) agreed with that from: the cancer cell fraction in 82% of patients (18/22); the immune cell fraction in 68% (13/19); the normal brain cell fraction in 46% (6/13). We inspected the fold change in expression of LE50 genes, which are key for determining responder subtype, in the full tumor pseudo-bulk and the purified cell fractions. The results (Fig. 3B–D) show that LE50 gene expression dysregulation, post-treatment, is often in opposing directions in the cancer cell fraction versus the normal brain cell fraction and is not observed in the immune cell subset. Hence, whilst the cancer cells are driving the responder subtype phenomenon, subtype calling is affected by normal brain cell infiltration. In the 4 cases where the responder subtype determined from the full tumor pseudo-bulk did not agree with the purified cancer cell pseudo-bulk, it did agree with the normal brain cell pseudo-bulk derived subtype, suggesting that this cellular component had driven the delineation. Further investigation showed that both tumors in the pair need to have a tumor purity of > 30% to be confident that the responder subtype call is that of the cancer cell subset (Fig. 3E). We applied GBMdeconvoluteR to the full tumor pseudo-bulk profiles and, using linear regression, found that the resulting scores can be formulated to accurately predict the true tumor purity (coefficient of determination, *R*^2^ = 0.78; Fig. 3F). We, therefore, used the GBMdeconvoluteR scores for the Discovery and Validation cohort samples to predict the purity of those tumors and found that all but one had tumor purity > 30% (Fig. 3G). Hence, these cohort samples can be used to further probe the biology underpinning the differential treatment response intrinsic to the cancer cells in Up versus Down responders.

### Genes that are differentially expressed post-treatment in the responder subtypes suggest different adaptive treatment resistance mechanisms

**Fig. 4:**
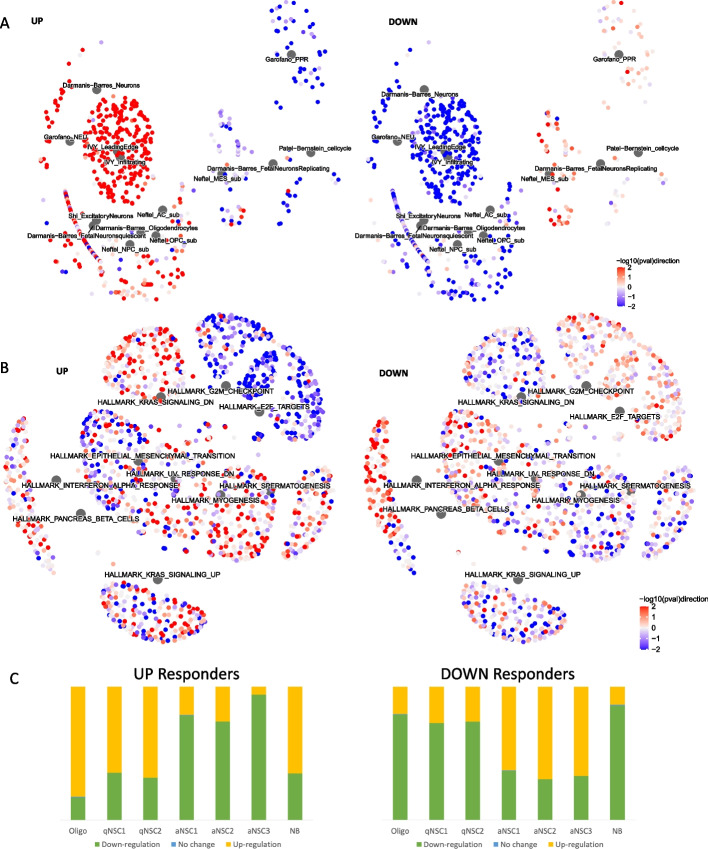
**A** Network plots showing the GBM biology-specific gene sets (described in Additional file 1: Table S24) that are significantly enriched (FDR < 0.05) in either, or both, Up or Down responders through treatment. Large gray hub nodes indicate the gene sets. These have associated, smaller, leaf nodes signifying the genes in that set, colored according to the strength and direction of differential expression through treatment (quantified as -log_10_p-value multiplied by the direction of fold change: -log10(pval)direction) in Up responders (left image) or Down responders (right image). **B** As for panel C but visualizing the Hallmark gene sets from MSigDB that were enriched with an FDR < 0.25 in either, or both, responder subtypes. **C** The proportion of neural stem cell markers of quiescence (qNSC) or active cycling (aNSC), or markers of more differentiated neuroblasts (NB) or oligodendrocytes (Oligo), that were upregulated (yellow), downregulated (green), or stable (blue) in Up (left) and Down (right) responders

We proceeded, using our bulk RNAseq data, to perform gene set enrichment analysis on the genes differentially expressed between primary and recurrent GBMs in the responder subtypes separately, to identify distinct biological processes occurring over time. To probe brain and GBM biology more specifically, we collated custom gene sets, alongside those from the Molecular Signatures Database (MSigDB), from the literature and public databases [3, 5, 11–33]. These included the latest cell type markers from the BRAIN Initiative Cell Census Network, and phenotypic GBM gene signatures from recent single-cell studies (Additional file 1: Table S24). Signatures of neuronal (NEU) neoplastic GBM cells, as defined by a study inspecting distinct pathways activated in single cell clusters [32], were significantly upregulated in Up responders (90% of genes in this set, Garofano_NEU, were significantly upregulated; FDR = 0, *n* = 40; Fig. 4A and Additional file 1: Table S25). This GBM cell type has elevated levels of neurotransmitter receptors implicated in glioma-neuron interactions, including synapses. In Up responders, gamma-aminobutyric acid (GABA) neurotransmitter signaling components, specifically, were significantly upregulated (85% of GABA-gated chloride ion channel activity genes were significantly upregulated through treatment in Up responders *n* = 13 FDR = 0.03 and downregulated in Down responders, FRD = 0.0053; Additional file 1: Table S26). Gene signatures of specifically differentiated type of neurons, oligodendrocytes, and the infiltrative leading edge from patient GBM samples were also significantly upregulated in Up responders (FDR < 0.05). All of these gene sets were significantly downregulated in Down responders (Fig. 4A and Additional file 1: Table S25). This suggests increased neuronal signaling and interactions between cancerous and normal brain cells through treatment in Up responders, specifically, alongside genes that demarcate processes of neuronal and glial cell differentiation.

Signatures of developmental glioma stem cell (GSC) states were also upregulated in Up, and downregulated in Down, responders (Richards_Developmental gene set, *n* = 444, FDR = 0.10 and 0.07, respectively; 94–97% of DE genes being unidirectionally dysregulated) and, specifically, signatures of oligodendrocyte progenitor cell-like (OPC) neoplastic cells, in agreement with the cell deconvolution results (Neftel_Cell_2019_OPC gene set, *n* = 49, FDR = 0.11 and 0.05, respectively; 93–100% of DE genes being unidirectionally dysregulated; Figs. 2A and 4A and Additional file 1: Table S25). Wang et al. [9] performed single-cell transcriptional analysis of GBM tissues, showing that cells can be transcriptionally assigned along a proneural GSC (pGSC) to mesenchymal GSC (mGSC) axis, with more differentiated malignant cells in the middle [5]. pGSCs uniquely expressed OPC genes. Together with the bulk tumor subtyping results, this suggests that Up responders become enriched for pGSCs through treatment. Conversely, in Down responders, there is repeated enrichment of mesenchymal GBM cell signatures (Figs. 2A and 4A and Additional file 1: Table S25). Within the MSigDB gene sets, there is also enrichment for genes associated with epithelial to mesenchymal transition. These genes are significantly more downregulated in Up responders and upregulated in Down responders (adjusted chi-squared *P* = 1.36E − 5; Fig. 4B and Additional file 1: Table S27). In GBM, a non-epithelial cancer, this hallmark process is more accurately referred to as proneural to mesenchymal transition [34]. Across both the MSigDB and custom gene sets, there was evidence that Down responders upregulate cell cycle genes from primary to recurrent, whereas these are being decreased in Up responders (Fig. 4A, B and Additional file 1: Tables S25-27). Conversely, in Up responders, there is significant upregulation of neural stem cell (NSC) quiescence markers. Codega et al. extracted NSCs from adult mouse brains, used label retention approaches to separate those that were quiescent (qNSCs) from those that were activated (aNSCs), and identified differentially expressed genes [31]. We found that a significant number of qNSCs markers were upregulated in Up responders (73%, *n* = 331; NES = 1.2, FDR = 0.10; Additional file 1: Table S25) with most of the same genes downregulated in Down responders (73%, *n* = 327; NES = 1.2, FDR = 0.07; Additional file 1: Table S25). Llorens-Bobadilla et al. did scRNAseq of murine adult subventricular zone NSCs, isolated via expression of GLAST and Prom1, and identified 7 unsupervised clusters: two defining qNSCs, three defining aNSCs, one defining neuroblasts (NB), and one defining oligodendrocytes (Oligo; which express low levels of both NSC marker proteins) [30]. We included the genes that delineated these clusters in our custom gene sets (Additional file 1: Table S24). We found that Up responders upregulate markers of both qNSCs clusters and the more differentiated NB and Oligo cell types, with concomitant downregulation of genes for all three aNSC clusters, whereas the opposite was evident in the Down responders (Fig. 4C and Additional file 1: Table S25).

### Gene expression correlation networks highlight different genes influencing transcriptional reprogramming in responder subtypes

**Fig. 5:**
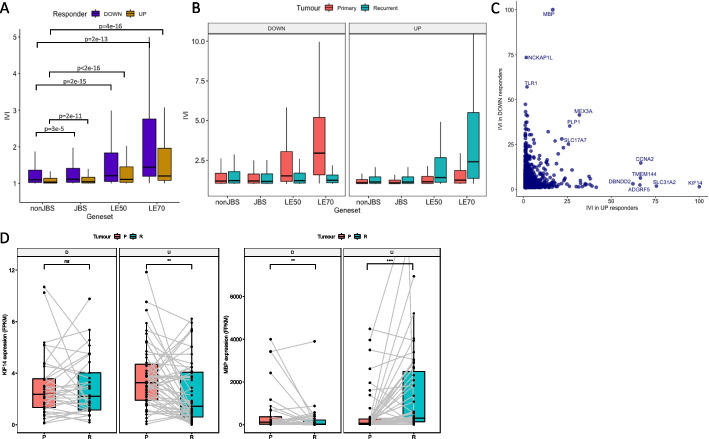
**A** The distribution of integrated value of influence (IVI) scores for different gene sets, calculated from log2FC (fold change in expression from recurrent to primary) correlation networks, for Down (purple) and Up (gold) responders. nonJBS: genes not in the JARID2 gene set; JBS: genes in the JARID2 gene set but excluding those in the leading edge of at least 50% of patients (LE50 genes); LE50: genes in the LE50 gene set but excluding those in the LE70 gene set; LE70: genes in the leading edge of at least 70% of patients. **B** As panel **A** except correlation networks were built from gene expression data in primary (salmon pink) or recurrent (teal) tumors in Down (left panel) or Up (right panel) responders, separately. **C** Genes are plotted according to their IVI score in log2FC networks of Down (*x*-axis) and Up (*y*-axis) responders. Genes that are high in both, or uniquely high in one, log2FC network are labeled. **D** The expression values for genes encoding Kinesin Family Member 14 (KIF14: left image) and Myelin Basic Protein (MBP: right image) are shown in the primary (salmon pink) and recurrent (teal) tumors of Down (D) and Up (U) responders. Gray lines indicate expression values in primary and recurrent GBMs from the same patient. Significance is denoted: ns, not significant; *p < 0.05; **p < 0.01; ***p < 0.001; ****p < 0.0001

To investigate what was driving transcriptional reprogramming in opposing directions, we performed parsimonious gene correlation network analysis (PGCNA), creating networks based on the fold change in expression from primary to recurrence (Log_2_FC) in Up and Down responders separately. We calculated the integrated value of influence (IVI) for each gene in each network. IVI summarizes numerous network parameters, such as hubness and degree centrality, to provide a metric of the overall importance of each gene in a network. We investigated the distribution of IVI among non-JBS, JBS, LE50, and LE70 genes and found that the leading-edge genes (LE50 and, even more so, LE70) are significantly more influential with regard to longitudinal gene expression dysregulation (Fig. 5A). We then built separate gene expression networks, following batch correction (Additional file 2: Fig. S3), for Up responder primary samples (Primary Up), and for Primary Down, Recurrent Up, and Recurrent Down. LE50 and LE70 genes are significantly more influential in primary samples of Down responders and in recurrent samples of Up responders (Fig. 5B). These changes are observed across several local and global parameters that quantify gene regulation within each network: spreading, hubness, betweenness, and degree (Additional file 2: Fig. S4). This implicates the most commonly dysregulated JBS genes, specifically, in driving the whole transcriptome reprogramming observed through treatment, and further validates that this reprogramming occurs in opposing directions.

Comparing individual IVI values in the Up vs Down responder Log_2_FC networks highlighted genes that were uniquely important in coordinating transcriptional reprogramming for each subtype (Fig. 5C). Kinesin Family Member 14 (*KIF14*) is highly influential in the Up responders only (IVI is 100 in Up and 1.4 in Down responders, Fig. 5C). This gene does not change expression between paired primary and recurrent samples in Down responders but significantly decreases in expression in Up responders (adjusted *P* = 2.0E − 3, Fig. 5D). KIF14 is a microtubule motor protein with a role in cell proliferation. Myelin Basic Protein (*MBP*) is highly influential in the Down responders only (IVI is 100 in Down and 16.7 in Up responders, Fig. 5C). *MBP* is significantly downregulated through treatment in Down responders and also significantly upregulated in Up responders (adjusted *P* = 8.9E − 3 and 2.70E − 10 respectively Fig. 5D). MBP is a major constituent of the myelin sheath of oligodendrocytes. This suggests that the genes that are uniquely influential in longitudinal transcriptional reprogramming in each responder subtype are so because they become downregulated. Their influence may, therefore, be exerted by becoming rate limiting to the specific processes they are part of, which we showed above to be associated with the opposing responder subtype. This implies that reducing the expression of key genes identified through these network analyses could prohibit certain adaptive mechanisms.

### JBSgenes are hypomethylated and significantly associated with the PRC2 histone mark: H3K27me3

**Fig. 6:**
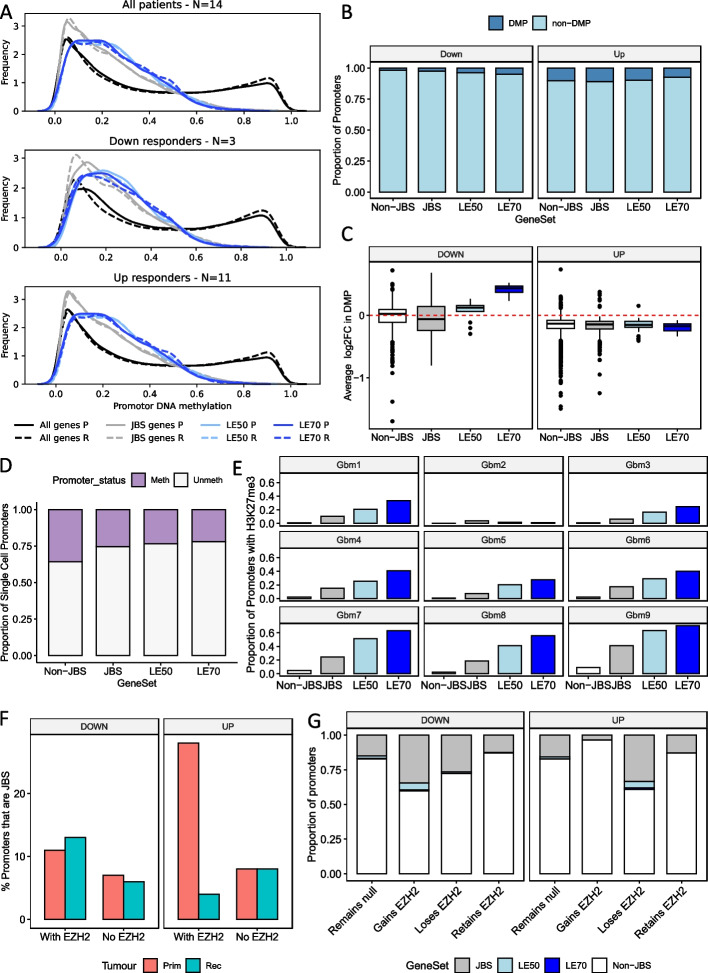
**A** The distribution of average promoter DNA methylation for all genes, JARID2 binding site (JBS)genes, LE50 and LE70 genes in primary (P) and recurrent (R) GBM tumors from all patients (top) and once separated into Down (middle) and Up (bottom) responders. **B** The proportion of differentially methylated promoters (DMP) between primary and matched recurrent GBM. **C** The average change in methylation between primary and matched recurrent tumors for the DMP from panel **B**. **D** The proportion of single GBM cell promoters that have different methylation status. **E** The proportion of promoters with the H3K27me3 mark in nine patient GBMs. **F** The proportion of JBSgene promoters that had EZH2 bound according to ChIPseq of paired patient samples from an Up and a Down responder. **G** The proportion of promoters with a specific change in EZH2 occupancy that belonged to each gene set

Having established that Up and Down responders have differential transcriptional reprogramming between their primary and recurrent GBM, we wanted to further inspect the potential underpinning mechanism. DNA methylation profiles are significantly altered in brain cancers, including GBM, and methylation of key genes has been shown to impact GBM standard treatment response [35–37]. There was DNA methylation data available for 14 of the longitudinal GBM pairs in our study: 11 Up and 3 Down responders [38]. JBSgene promoters, and more specifically those of the LE50 and LE70 genes, are hypomethylated in both primary and recurrent tumors, including when separated into responder subtype (Fig. 6A). The proportion of differentially methylated promoters (DMPs) between paired primary and recurrent tumors is similar for when comparing Non-JBS and JBS promoters, irrespective of responder subtype (Fig. 6B). Up responders do, however, have a higher proportion of DMPs (7–10%) than Down responders (2–5%; Fig. 6B). In the minority of promoters that are DMP, the direction of change in methylation is consistent with the observed changes in genes expression per responder subtype (Fig. 6C). The level of single cell promoter DNA methylation, investigated using data from 5 patient GBMs from Johnson et al. [7] agrees with our bulk tissue findings, i.e., significantly fewer JBS, LE50, and LE70 gene promoters are methylated in the single cell data (chi-squared *P* = 0 for all pairwise tests; Fig. 6D). Collectively, these results suggest that differential DNA methylation is not the key driver of the bidirectional changes in gene expression observed in patients between primary to recurrence.

JARID2 binding sites are most significantly enriched in the promoters of the dysregulated genes in GBM but in the cancer cells, specifically, there is also enrichment of the catalytic subunit of PRC2: Enhancer of zeste homolog 2 (EZH2; Figs. 3A). This suggests that PRC2, and its associated histone modification, H3K27me3, could be key players in transcriptional reprogramming in GBM. We, thus, determined the H3K27me3 status of promoters in 9 GBM tumors that had undergone ChIPseq [39] and found that JBSgene promoters contain significantly more H3K27me3 than non-JBS genes in all 9 tumors (chi-squared tests *P* = 0; Fig. 6E). We also found that, for all tumors except GBM2, LE50 promoters contain significantly more H3K27me3 than JBS, and LE70 significantly more than LE50, promoters (chi-squared tests *P* < 0.01; Fig. 6E). To ascertain the change in PRC2 occupancy at different promoter sets between primary and recurrent tumor, we performed EZH2 ChIPseq in longitudinal samples from one Up and one Down responder from our cohort. In the Down responder, there were 1.4 × more EZH2-bound JBS promoters in the primary sample than expected given the occupancy at non-JBS gene promoters (chi-squared test *P* = 0), and this increased to 2 × more (chi-squared test *P* = 0) in the matched recurrence (Fig. 6F). Conversely, whilst in the primary tumor of the Up responder there was significant enrichment of EZH2 binding at JBS promoters (3.5 × higher; chi-squared test *P* = 0), in the matched recurrence, there was significantly less EZH2 binding than expected (2 × less occupancy; chi-squared test *P* = 0; Fig. 6F). When classing each promoter according to the change in EZH2 status between the primary and matched recurrence, we found that JBS genes are significantly more likely to gain EZH2 in the Down responder (chi-squared tests *P* = 0), but to lose EZH2 in the Up responder (chi-squared tests *P* = 0, *P* = 8.6E − 16, and *P* = 2.0E − 8 for comparison between non-JBS and JBS, LE50 or LE70 respectively; Fig. 6G). These results suggest that the genes implicated in longitudinal transcriptional reprogramming in GBM are regulated by PRC2 via the repressive mark H3K27me3.

### Responder subtypes are recapitulated in preclinical models of GBM, but tumor microenvironment is required

**Fig. 7:**
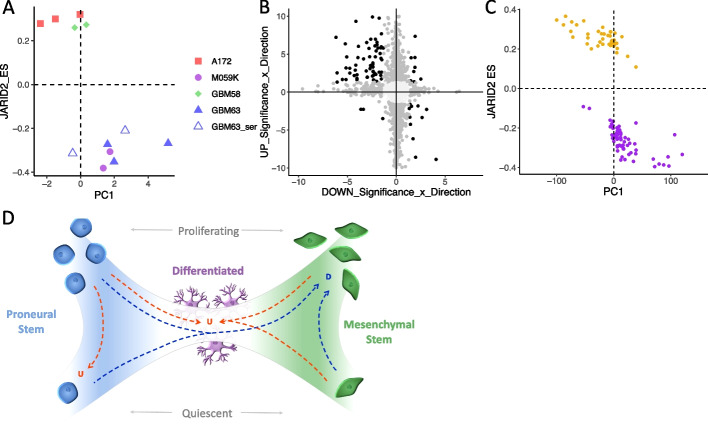
**A** Replicate experiments in GBM cell line spheroids with and without chemoradiation are plotted according to the JARID2 gene set enrichment score (ES) and the value of first principal component (PC1) when results are projected onto the patient principal components in Fig. 1E. **B** Results of differential expression (DE) analysis between treated and untreated spheroids of Up responder and Down responder cell lines separately (*n* = 2 or 3). Genes are plotted according to their -log_10_-adjusted *p*-value multiplied by the log_2_fold change (FCp). Colors denote if the gene is significantly DE (FDR < 0.05) in none (light gray), one (dark gray), or both (black) responder subtypes. **C** Patient GBM samples cultured as organotypic slices and either treated with irradiation and TMZ or left untreated plotted according to the JARID2 gene set ES and PC1 when results are projected onto the patient principal components shown in Fig. 1E. Models are colored according to whether they are Up (gold) or Down (purple) responders. **D** Our working model to explain GBM responder subtypes: GBM cells are on a phenotypic axis between proneural and mesenchymal stem cells. These stem cells can be in a quiescent or actively cycling state. Differentiated, interconnected (with both each other and surrounding normal cells) cell states lie in the center of the axis. In Down responders, cells in the GBM tumor move towards the mesenchymal phenotype and increase proliferation rates over time. In Up responders, neoplastic GBM cells either become more differentiated and integrate with surrounding cells, upregulating neurotransmitter signaling as they do, or they convert to or remain as proneural stem cells but in a quiescent state over time

Our findings indicate that GBM tumors can be stratified by differential transcriptional reprogramming from primary to recurrence, implicating subtype-specific mechanisms of treatment resistance that could yield subtype-specific therapeutic targets. To test this requires experimental models that recapitulate responder subtypes. Such models would also enable investigation of the epigenetic mechanisms underpinning transcriptional reprogramming without the limitations imposed by the reduced availability and amount of patient tissues for such studies. Our single-cell analyses show that the responder subtype is a cancer cell-intrinsic property, so we first assessed GBM cell lines in vitro*.* We cultured established (A172 and M059K) and patient-derived, serum-free (GBM58 and GBM63) GBM cell lines as 3D spheroids and subjected them to physiologically relevant, non-surgical elements of standard treatment: 2 Gy irradiation and 30 μM TMZ. We also performed this experiment on GBM63 cell lines cultured in serum, which causes them to become more differentiated (denoted GBM63_ser). Treatment caused a significant reduction in spheroid size in all cell lines in comparison with untreated controls (average spheroid size reduced in treated samples by 17.45%: *t*-test, *P* = 5.2E − 3 for A172; 20%: *t*-test, *P* = 8.28E − 34 for M059K; 9%: *t*-test, *P* = 2.09E − 7 for GBM58; 18.11%: *t*-test, *P* = 1.0E − 4 for GBM63; and 21%: *t*-test, *P* = 1.07E − 21 for GBM63_ser). At the endpoint, RNA was extracted for each experiment (*n* = 2 for M059K, GBM58, and GBM63_ser, and *n* = 3 for A172 and GBM63). Plotting the fold change in expression between treated and untreated samples (Log2FC) according to the JARID2 enrichment score (ES) and the first principal component (PC1) separated these cell lines as it did for patient tumors (Figs. 1H and 7A). This denoted A172 and GBM58 as Up, and M059K and GBM63 as Down, responders; we note that the addition of serum did not alter the responder subtype of GBM63 (Fig. 7A). Differential gene expression corroborated the different direction of transcriptional dysregulation in these cell lines in response to treatment: 84 out of 97 (87%) genes DE in both responder subtype cell lines were dysregulated in opposite directions (Fig. 7B). However, we note that the range of values for PC1 for cell lines (Fig. 7A) is ~ 100 × smaller than we observe in patients (Fig. 1H). PC1 represents the main source of variance in gene expression between treated/recurrent and untreated/primary samples, making it a proxy for the extent of transcriptional reprogramming. Our results, therefore, suggest that 3D cell line models do recapitulate responder subtypes, but the effect size differs from that in patients.

We reasoned that this is because of a lack of TME components in our model system. To test this, we acquired data from a study where organotypic GBM slices from 25 patients were cultured and treated with irradiation (4 Gy) and TMZ (200μM) ex vivo, or left as untreated controls, before undergoing RNA sequencing [40]. The ex vivo model system retains the tissue architecture of the patient tumors, allowing us to investigate whether the act of cell culture, alone, affects the extent of transcriptional reprogramming connoted by PC1. We found that plotting the ex vivo models by their JARID2 enrichment score (ES) and the first principal component of all-gene Log2FC (PC1) separated them into Up and Down responders with a PC1 range more akin to that seen in vivo in patients (Fig. 7C).

## Discussion

We have shown that a subset of genes is consistently and significantly dysregulated in locally recurrent IDHwt GBM tumors, following standard treatment, but that the direction of dysregulation is patient-specific. This phenomenon classifies patients into Up or Down responders (Fig. 1D and Additional file 2: Fig. S1C). Single-cell analysis shows that the responder subtype is a cancer cell-intrinsic property, but that the gene dysregulation is also observed in the normal brain cell component of the TME in the opposing direction (Fig. 3B–D). The purity of the tumor, thus, impacts whether the subtype call is driven by the cancer cell fraction, with purity > 30% required (Fig. 3E).

Inspecting the post-treatment transcriptional changes in responder subtypes separately potentially suggests different mechanisms of treatment resistance. However, we note that our study does not include patients who have not received treatment, so we cannot conclude that the transcriptional reprogramming we see is therapy-driven. This exclusion was pragmatic: surgery is the main component of treatment and the means by which samples are acquired so cannot be controlled for; the majority of patients receive chemoradiation or at least one component thereof (particularly those subsequently deemed suitable for a recurrent surgery), meaning there are too few patients to perform a meaningful comparative study pertaining to this aspect of standard therapy. It should also be noted that, herein, the term “recurrent” does not always equate to tumor regrowth: the surgical interval for some patients in our study suggests a second operation on the remaining primary tumor. In all cases, though, the recurrent tumor is post-treatment (following surgery and chemoradiation) so constitutes cells that have evaded killing by multi-modal therapy. Notwithstanding these considerations, we see clear, differential longitudinal gene expression changes that stratify patients and allude to distinct modes of tumor development over time, which could be therapeutically targetable.

In Down responders, we observe a predisposition of tumors to become more mesenchymal, both at the bulk level and via an increase in mesenchymal neoplastic cells specifically (Fig. 2A–C and Additional file 2: Fig. S2A). Down responders also exhibit upregulation of proliferation and cell cycling (Fig. 2B, C and Additional file 1: Table S4 and S6). In keeping with this finding, Wang et al. [9] recently showed an increase in the number of cycling single GBM cells, of the mesenchymal state specifically, at recurrence. The mesenchymal phenotype has been linked with intrinsic resistance to both radiation and TMZ in gliomas [41–43]. This suggests that, in Down responders, there is a selection of, or transformation to, more inherently resistant mesenchymal cells post-treatment, with subsequent expansion to form a recurrent tumor.

Conversely, in Up responders, we observe a higher probability of switching towards a proneural subtype (Fig. 2A, B), driven by an increased prevalence of neural- and, especially, oligodendrocyte-progenitor-like cancer cells at recurrence (Figs. 2C and 4A and Additional file 2: Fig. S2A). The study by Wang et al., whilst concluding that there was an overall shift towards a mesenchymal phenotype in single cells, clearly showed a subset of tumors with increasing proneural fractions at recurrence, as noted in the accompanying review article and further explained by our realization of the need for patient stratification [9, 44]. In this subgroup, we observe a reduction in cell cycling and upregulation of quiescence (Fig. 4A–C and Additional file 1: Table S25 and S27). Up responders also exhibit a longitudinal shift towards more differentiated cell states, a unique upregulation of neuronal signaling, and upregulation of signatures observed within the leading edge of GBM tumors which is more enriched with normal brain cells (Fig. 4A and Additional file 1: Table S25 and S27). GBM cancer cells form synapses with surrounding neurons and glia, creating circuits through which electrical signaling has been shown to promote glioma growth [45–47]. Radiation and TMZ target rapidly dividing cells. Our results suggest that, in Up responders, cells might evade treatment by reducing proliferation through either quiescing or becoming more differentiated and integrating into normal neural circuits. Future work will focus on greater interrogation of the specific types of brain cells, and details of neurotransmitter signaling, involved.

Our previous work spanning all types of longitudinal glioma concluded that there are recurrent phenotypes: neuronal, mesenchymal, and proliferative, with the former two being specific to IDHwt tumors [8]. Herein we have confirmed these phenotypes but shown that patients can actually be stratified according to phenotype shift at recurrence: Up responders become more neuronal and Down responders become more mesenchymal and proliferative. This suggests that tumor progression over time is polarizing, as also confirmed by Wang et al. [9] who initially defined the proneural to mesenchymal axis in patient tissues. Their most recent work shows that tumors become more polarized to either end of this axis over time during treatment [9].

Cumulatively, this has led to our working model of GBM tumor adaption or development, detailed in Fig. 7D. We propose that Down responder tumors (blue dotted arrows) convert to a more proliferative, mesenchymal state that is able to continue dividing owing to being more intrinsically able to survive standard treatment; Up responders (red dotted arrows) reduce proliferation, thus avoiding death by radiation and TMZ, by converting to a more quiescent, proneural phenotype or becoming more differentiated and able to integrate with normal neural circuits. If not relevant to treatment resistance, these phenotypic changes may suggest differential intrinsic requirements to develop interactions with varying components of the TME as the tumor progresses. In either case, our results raise the possibility that responder subtype stratification will lead to more effective, targeted treatments.

An alternative therapeutic approach to targeting the biological differences downstream of responder stratification is to target the mechanism by which responder subtypes derive, i.e., the mechanism that underpins reprogramming in different directions. Such a target may be universal. To begin assessing this, we first investigated DNA methylation patterns in individual tumors and longitudinal pairs. We found that the dysregulated genes have mostly unmethylated promoters and that only a minority were differentially methylated longitudinally (Fig. 6A, B, and D). This is in agreement with other longitudinal studies that showed significant methylation stability between matched primary and recurrent GBMs [38, 48], implying that alterations in DNA methylation do not drive the observed longitudinal gene expression changes. Our data implicates JARID2, as the dysregulated genes were identified through commonality of binding this protein in public datasets (JBSgenes). JARID2 is an accessory protein to Polycomb Repressive Complex 2 (PRC2), an epigenetic remodeling complex with known roles in brain cell lineage determination. PRC2, via its catalytic subunit EZH2, is responsible for the repressive histone mark H3K27me3. PRC2 has been implicated in several recent GBM studies aimed at understanding modes of GBM cancer cell plasticity at the bulk and single-cell level [6, 48]. Our data shows enrichment of H3K27me3 at JBSgene promoters in patient GBMs (Fig. 6E) and significant association of, and longitudinal changes in the binding profile of, EZH2 at JBSgenes in ways that differ depending on responder subtype (Fig. 6F, G). This supports the notion that histone-based remodeling is responsible for driving longitudinal changes. To confirm this requires histone and PRC2 profiling in a larger cohort of longitudinal samples, but this is a considerable challenge owing to the amount of fresh (frozen) tissue needed for such approaches and the rarity of such samples.

The bidirectional nature of subtype transcriptional reprogramming suggests that adjuvant therapies that benefit one subtype could be detrimental to the other. This may explain previous failures of clinical trials. Retrospective subtyping could elicit whether certain therapies would be more effective post-stratification but to test whether patient stratification could yield more effective treatments in earnest, we must identify suitable experimental models for target validation and drug screening. Having models that recapitulate the responder phenotypes observed in patients will also (1) enable a more robust and comprehensive evaluation of the epigenetic mechanisms responsible and (2) facilitate dissection of whether and which aspects of treatment are associated with the phenomenon. We identified that both established and patient-derived GBM cell lines can be assigned to responder subtypes, based on transcriptional reprogramming in response to treatment (Fig. 7A, B). However, the extent of transcriptional reprogramming was orders of magnitude smaller than we see in patients. Using data from GBM organotypic slice cultures, we found that retention of tissue architecture and the TME increases the level of transcriptional reprogramming to align more with patients, despite being cultured in media and treated ex vivo*.* A recent study in an extensive panel of cell lines revealed a profound redistribution of histone marks in GBM, compared to normal brain astrocytes and OPC, which was directly associated with altered gene expression, including specifically of neural genes and those involved in neurogliomal synapse formation [49]. Inhibition of SMAD3, one of the factors implicated in the pathological rewiring of the GBM cell lines, was shown to only yield phenotypic results when cell lines were co-cultured with neurons [49]. These results align with our findings and suggest that experimental systems must incorporate the TME in order to adequately model responder phenotypes. Identifying suitable models will be the focus of future work.

## Conclusion

Our work has identified two responder subtypes in GBM, suggesting that previously identified recurrent phenotypes result from the adaption of tumors along a phenotypic axis. The mechanism enabling this adaption, or the biology underpinning the differential responses, yields the opportunity for adjuvant treatments to make the standard of care more effective for patients with this deadly disease.

## Methods

Data analyses were performed in R [50] and Python 3 with plots made using ggpubr [51].

### Sample collection and processing

Longitudinal GBM samples were acquired from The Walton Centre, Lancashire Teaching Hospitals, and Leeds Teaching Hospitals National Health Service (NHS) Foundation Trusts via the Brain Tumour Northwest Tissue Banks and the Leeds Neuropathology Research Tissue Bank. In addition, tissue samples were obtained from Cambridge University Hospitals NHS Foundation Trust as part of the UK Brain Archive Information Network (BRAIN UK) [52]. Samples were processed as previously described [53]. Briefly, formalin-fixed paraffin-embedded (FFPE) blocks were sectioned and the first and last sections were H&E-stained and underwent neuropathologist review to identify areas of > 60% tumor. Regions of overlap were macrodissected from the intervening sections and RNA was extracted using AllPrep DNA/RNA FFPE Kit (catalogue #80,234) from Qiagen (UK). Fresh frozen longitudinal GBM samples were sent to Active Motif (Carlsbad, CA) for ChIP-Seq analysis.

### RNAseq data acquisition and processing

All RNA extracted in-house underwent rRNA depletion using the NEBNext rRNA Depletion Kit (Human/Mouse/Rat) and then strand-directional, whole transcriptome library preparation using NEBNext Ultra™ II Directional RNA Library Prep Kit for Illumina®, both from New England Biolabs (UK). Libraries were sequenced on Illumina next-generation sequencers as 100 bp paired-end reads. Raw RNA data was acquired from several published studies following the negotiation of Data Transfer Agreements, where necessary [2, 8, 54–56] (see Additional file 1: Table S1). All Discovery cohort, and in vitro, FASTQ data were trimmed of low-quality bases, Phred threshold = 20, and adapters via Trim Galore v0.4.3, wrapping Cutadapt v1.8.3 [57]. Trimmed reads were quality checked using FASTQC [58] and then aligned to the human reference genome GRCh38.13 using STAR v020201 in two-pass mode with a maximum of 5 multireads [59]. Gene and transcript count and gene expression were quantified via CuffQuantv2.2.1 taking directional specifics of the library as input, using probabilistic weighting of multireads and quantifying against the GENCODEv27 human genome annotation with haplotypes and scaffolds included [60, 61]. Sequencing metrics are given in Additional file 1: Table S2. Validation cohort data (Additional file 1: Table S1) was acquired as pre-processed transcript counts and transcripts per million (TPM) via the GLASS portal at https://www.synapse.org/glass [8]. Transcript expression was converted to gene-level or transcription start site (TSS)-level data by summing isoform expression. Genes were filtered to keep only those that were expressed above the lower quartile of non-zero gene expression in at least 25% of either primary or recurrent samples.

### Differential expression analysis

Differential expression (DE) analysis was performed using DESeq2 using a paired design [62]. Functional enrichment was assessed via WebGestalt (v2019) [63] with gene set size < 1000 on DE genes with FDR < 0.01. The DE enrichment diagram was created using enrichplot R package [64]. The statistical significance of the overlap between differentially expressed genes from different cohorts was inferred using the hypergeometric method as implemented in http://nemates.org/MA/progs/overlap_stats.html. DE was also run separately on Up and Down responders with results given in Additional file 1: Tables S22-23. To evaluate differences in the directions of dysregulation of gene sets between responder types, 2 × 2 contingency chi-squared tests were performed on the numbers of genes that significantly increased or decreased through treatment in Up and Down responders, in addition to 2 × 3 chi-squared tests on the numbers that increased, decreased, or remained stable. The raw *p*-values as well as adjusted FDRs are given alongside GSEA results in Additional file 1: Tables S25-27.

### Gene set enrichment analysis

We developed a novel gene set file for use in GSEA using the Gene Transcription Regulation Database (GTRD v19.10) [65]. A gene was assigned to a DNA-binding factor’s gene set if its promoter (transcription start site from gencodev27 ± 1kbp, or ± 2 or 5kbp where specifically stated) contained a binding site for that factor in ≥ 2 independent ChIPseq experiments. We first performed pre-ranked GSEA [66], per patient, ordering genes by the magnitude of fold change in expression log_2_(|recurrent expression + 0.01/primary expression + 0.01|) in classical mode. To indicate the directionality of dysregulation, we then ranked genes by fold change, referred to throughout as Log_2_FC, i.e., using log_2_(recurrent expression + 0.01/primary expression + 0.01), and weighted by magnitude [67]. Cell lines were processed the same as the above, with arbitrarily paired untreated and treated replicates representing primary and recurrent samples. When running GSEA on Up and Down responders separately, genes were ranked and weighted based on the log2 of their significance in differential expression from primary to recurrent. All GSEA runs were set for 1000 permutations and to allow gene set sizes of up to 50,000. Results are given in Additional file 1: Tables S3-18. Heatmaps were created using ComplexHeatmaps R package [68]. Gene set network diagrams were created using enrichplot R package [64].

### Batch correction

Each pair of samples was included in the same batch for processing and profiling, such that log2FC-based analyses did not require prior batch correction. However, to enable analysis of single samples, we performed batch correction following the approach detailed in Fig. S3. We removed genes with zero count across all samples, removed non-protein coding genes (as not all library prep methods captured full transcriptomes), and applied ComBat-Seq [69]. We visualized batches before and after correction, to ensure no further clustering by contributing center was evident, using PCA, t-SNE, and UMAP via the M3C R package, v1.10.0 [70].

### Tumor subtyping and cell deconvolution

GBM subtype calls were performed using GlioVis [71] with the 3-way assignment used. Sankey plots were created with SankeyMATIC https://sankeymatic.com. IDHwt GBM tumor-specific cell deconvolution was done using GBMDeconvoluteR using the Ajaib et al. gene markers, supplemented with cells for which only Ruiz-Moreno et al. gene markers were available, with non-tumor intrinsic genes filtered out [26, 72, 73].

### Survival analysis

A multivariate linear regression was performed to assess the relation between progression-free and overall survival (months) and the explanatory variables: age (years), batch-corrected MGMT expression (transcripts per million) in the primary tumor, and JARID2 gene set normalized enrichment score. Data were checked for multicollinearity with the Belsley-Kuh-Welsch technique. Heteroskedasticity and normality of residuals were assessed respectively by the Breusch-Pagan test and the Shapiro–Wilk test. A *p-*value < 0.05 was considered statistically significant. Statistical analysis was performed with the online application EasyMedStat (version 3.16; www.easymedstat.com).

### Single nucleus RNAseq data analysis

snRNAseq datasets from were downloaded from GSE174554 and processed using Seurat’s SCTransform method (v4.3.0) [9]. Aneuploid cells were separated from diploid based on annotations from Wang et al. [9]. Diploid cells were further separated by clustering cells via Seurat’s FindClusters method, using the first 30 principal components, and classifying clusters as Immune cells if > 5% cells expressed PTPRC (CD45), or Non-immune diploid if not. Pseudobulk expression profiles were generated for each cell fraction (“Aneuploid,” “Immune,” “Non-immune diploid”) for each sample by first subsampling cell fractions so that paired primary and recurrent cell fractions contain equal cell numbers, and then getting the mean expression across cells. A fourth pseudobulk (“All”) was generated for all cells in the original bulk samples. Responder types were determined by running GSEA on the log2FC of the pseudobulks, as per the bulk RNAseq data. The true purity of each sample was calculated as the number of cancer cells as a fraction of all the cells sequenced. Purity estimates were acquired from GBMdeconvoluteR scores by performing each possible fraction with cancer cell scores summed on the numerator, and both cancer and non-cancer cells on the denominator. Each estimated purity was plotted against true purity and linear regression (optimization of the regression coefficient) was used to determine the final equation to use.

### Gene correlation networks

Log2FC values were used to build correlation networks for Up and Down responders separately with Parsimonious Gene Correlation Network Analysis (PGCNAv2) [74] using the Ledenalg algorithm for community detection. Default parameters were used except for setting “ –pgcna_f 1, –pgcna_n 1000” to ensure that all genes were used in network construction and that the clustering algorithm is run 1000 times to enable the optimal network to be assessed, and selected, according to the Scaled Cluster Enrichment Score (SCES). An integrated value of influence (IVI) score was assigned to each gene using the influential R package [75].

### DNA methylation data analysis

Bulk DNA methylation data was sourced from the GLASS Synapse page (filename beta.merged.tsv acquired via https://www.synapse.org/#!Synapse:syn23594913), having been processed as per Malta et al. 2021 [38]. The genomic map for each probe on an Illumina Infinium EPIC array 1.0 array was downloaded from Illumina (infinium-methylationepic-v-1–0-b5-manifest-file_extract.txt taken from https://emea.support.illumina.com/downloads/infinium-methylationepic-v1-0-product-files.html). Methylation data were filtered to contain probes overlapping promoters only for patients for which the responder subtype was known. Average promoter methylation values were plotted. Paired *t-*tests comparing average methylation between primary and recurrent samples were performed separately for each responder subtype and adjusted *p*-values < 0.05 were used to ascertain significance.

Single-cell promoter methylation data from Johnson et al. [7] were sourced from Synapse (https://synapse.org/singlecellglioma) as average beta values across all CpGs in a promoter (source file PromoterDNAmethylation.tsv). The data were filtered to only contain data from GBM tumors. Promoters were classed as methylated if they had a beta > 0.5, and unmethylated otherwise.

H3K27me3, and matched input control DNA, ChIPseq data for primary GBM samples was downloaded from the NIG Sequence Read Archive (Accession number PRJNA391756) in fastq format. EZH2, and matched input control DNA, ChIPseq data was created from samples in our Discovery cohort by Active Motif (Carlsbad, CA). Active Motif performed the chromatin preparation, ChIP protocols, library preparation, and library sequencing. Cells were dampened with 0.125 M glycine and fixed with 1% formaldehyde for 15 min. The fixed cells were mixed with lysis buffer and then agitated in a Dounce homogenizer. With the use of Active Motif's EpiShear probe sonicator (cat# 53,051), the resulting lysates were sonicated, and the DNA was sheared to an average length of 300–500 bp. To prepare the input sample, fractions of chromatin were treated with RNase and proteinase K. The mixture was heated to break down crosslinks. This was followed by SPRI beads cleanup (Beckman Coulter), and Clariostar quantification (BMG Labtech). With the use of protein G agarose beads, an aliquot of chromatin (50 μg) was precleared (Invitrogen). Four micrograms of Active Motif’s anti-EZH2 antibody was used to identify genomic DNA areas of interest. Complexes were cleaned, then treated with RNase and proteinase K after being eluted from the beads using SDS buffer. Crosslinks were broken down overnight at 65 °C, and ChIP DNA was then extracted using phenol–chloroform, followed by ethanol precipitation. SYBR Green Supermix was used in triplicate for quantitative PCR (qPCR) experiments on particular genomic regions (Bio-Rad). By running qPCR for each primer pair using input DNA, the signals were adjusted for primer efficacy. Illumina sequencing libraries were prepared using the conventional enzymatic procedures of end-polishing, dA-addition, and adaptor ligation. A robotic system (Apollo 342, Wafergen Biosystems/Takara) was used to carry out the steps. The resultant DNA libraries were measured and sequenced using Illumina's NextSeq 500 following a final PCR amplification step (75 nt reads, single end).

FASTQ data were trimmed of adapters and low quality (Phred score < 20) bases using cutadapt (v3.4), with reads only being retained if > 20 bp after trimming. Reads were aligned to the human reference genome (GRCh38.p13) using BWA MEM (v. 0.7.15-r1142-dirty). Duplicates were marked and removed using PICARD MARK DUPLICATES (v.2.6.0). Samtools (v.1.3.1) was used to index and sort bams and to remove any low-quality (mapping quality < 20) or secondary alignments, as well as unmapped reads. Reads mapping to ENCODE GRCh38 blacklist regions were removed (these regions were acquired from file accession ENCFF356LFX via https://www.encodeproject.org/files/ENCFF356LFX/). Promoters (TSS ± 1 kb) were called positive, i.e., containing a signal from histone methylation or protein binding, if they were assessed to have significant enrichment of reads in that region according to a method described previously [76, 77]. We calculate the average (λ) number of aligned reads (nr) in windows and the size of the regions of interest (*W* = 2kb) in our ChIP and control (input DNA) experiments. Windows were tiled, non-overlapping regions of size 2 kb. A read was counted if its midpoint was contained within the window.

$$\lambda_\text{input}=\frac{W\times{nr}_{input}\,}{genomeSize}$$

$$\lambda_\text{ChIP}=\frac{W\times{nr}_{ChIP}\,}{genomeSize}$$

Cancer genomes are not diploid and so changes in coverage are expected based on copy number aberrations, Hence, we use an adjustment term (input) to quantify, for any specific window (*w*), how much nr_input_ deviates from what is expected, given the genome average:

$$\varepsilon_{input}=\frac{{nrw}_{input}}{\lambda_{input}}$$

We assume that the number of reads in the ChIP experiment windows is expected to follow a Poisson distribution, $A$, in which.

If $$\varepsilon_\text{input}>1)\{A\sim\mathrm{Poisson}(\varepsilon_\text{input}x\lambda_\text{ChIP})\}$$  

Else $$\{ A \sim {\text{Poisson}}({\lambda }_{{\text{ChIP}}})\}$$

For each promoter window (p), we calculate the significance of the number of reads aligning therein in the ChIP experiment (${nrp}_{{\text{ChIP}}}=a$) using $P(A\le a)$ and then score promoters if FDR < 10^−15^. Three scripts were generated to perform these tasks: the “regioncounts” script, the “calculate-lambda” script, and the “calculate-promoter-signal” script.

### Preparation of stock solutions

Temozolomide (Merck, T577-100MG) was resuspended to 50 mM in dimethyl sulfoxide (DMSO) and stored at − 20 °C.

### Cell line acquisition and culture

The A172 and M059K established GBM cell lines were acquired from the ATCC (CRL-1620 and CRL-2365 respectively). A172 cells were maintained in DMEM (Merck, D6429) and 10% fetal bovine serum (FBS). M059K cells were maintained in DMEM/F12 (ThermoFisher Scientific, 11320033) supplemented with 10% FBS (ThermoFisher Scientific, 10270106), 0.5 mM NEAA (ThermoFisher Scientific, 11140050), and 1 mM sodium pyruvate (ThermoFisher Scientific, 11360070).GBM58 and GBM63, derived in Leeds, were cultured in NB media (ThermoFisher Scientific, 10888022) supplemented with 40 ng/mL recombinant human EGF (Peprotech, 236-EG-200), 40 ng/mL recombinant human FGF (R&D systems, 100-18B-100), 0.5 × B27 serum-free supplement (ThermoFisher Scientific, 17504044), and 0.5 × N2 supplement (ThermoFisher Scientific, 17502048). GBM58 and GBM63 were cultured in flasks coated with 10 μg/mL ornithine (Sigma, P3655-50MG) and 2 μg/mL laminin (Sigma, L2020-1MG). Cells were maintained at 5% CO_2_ at 37 °C and passaged when at 80% confluency. On passage of the cells, they were washed with 5-mL Dulbecco’s phosphate-buffered saline (PBS) before the addition of Trypsin–EDTA solution at 1 mL/75cm^2^ flask. Cells were placed in the incubator until detached before being collected in the appropriate media and centrifuged for 5 min at 300* g* before the media were removed. Cells were resuspended in the medium and split at appropriate confluency. For plate coating, poly-L-ornithine stocks were diluted to 10 μg/mL in TC-grade water. 10 mL working solution was added to each T75 flask. After 1 h at room temperature, the solution was removed, and flasks were rinsed with TC-grade water. Laminin stocks were diluted to 2 μg/mL in PBSA. 10 mL working solution was added to each T75 flask. Flasks and plates were wrapped in parafilm and left at room temperature overnight before storing at − 20 °C.

### Spheroid culture

For 3D culture, cells were trypsinized and resuspended at 1.5 × 104 cells per mL in normal culture medium supplemented with 10 mM taurine and 200 μL plated into each well of a 96-Well Clear Round Bottom Ultra-Low-Attachment Microplate (Scientific Laboratories Supplies, 7007). Any empty wells were filled with 200 μL PBS to avoid evaporation. Spheroids were imaged immediately before treatment and 1 week post-treatment using the Confocal Nikon AR1 and medium was changed every 3 days by removing 100 μL of medium and replacing this with 100 μL fresh medium.

### Treating with temozolomide and irradiation

At 5 days post-seeding, 100 μL of the medium was removed from each spheroid and replaced with 100 μL medium containing TMZ diluted from the 50 mM stock solutions to 60 μM, giving a final concentration when added to spheroids of 30 μM. One hour after TMZ addition, cells were irradiated using a RadSource RS-2000 X-ray irradiator with 2 Gy.

### Spheroid imaging and growth curves

To measure spheroid growth, a bespoke automated plate-imaging and analysis program was developed using the Confocal Laser Scanning Microscope-Nikon A1R. Area (μm^2^) would be the measurement used to represent spheroid size. Data was analyzed using SpheroidAnalyseR [78] which uses a pre-set threshold to remove obvious outliers for example empty wells, and then removes further statistical outliers using a robust *Z*-score of ± 1.96. Linear mixed effects models were built using R package lme4 v1.1.28 [79]. Effect sizes and significances were then calculated for the models fitted by maximum likelihood.

### RNA from spheroids for sequencing

Around 50 spheroids per condition were collected in a 15-mL centrifuge tube and centrifuged at 800 rpm for 5 min. Media was aspirated and spheroids were washed in PBS and centrifuged at 800 rpm for 5 min. PBS was removed and spheroids were washed in PBS and centrifuged at 800 rpm for 5 min. PBS was removed and 600 μL of Qiazol was added from the Qiagen Lipid Tissue Mini Kit. Spheroids in Qiazol were frozen at − 80 °C for 24 h before being defrosted. Once defrosted, Qiagen Lipid Tissue Mini Kit (Qiagen, 70,804) was used to extract RNA as per the manufacturer’s instructions. RNA was quantified using Nanodrop spectrophotometer (ThermoFisher) assessment before being stored at − 80 °C. RNA was sequenced and analyzed as per that from patient samples.

### Supplementary Information

**Additional file 1: Supplemental tables.** This file includes 27 supplemental tables of data pertaining to this study and referenced throughout the publication.

**Additional file 2: Supplemental figures.** This file includes 4 supplemental figures of data, along with accompanying figure legends, pertaining to this study and referenced throughout the publication.

**Additional file 3.** Review history.

## Data Availability

The raw data created for this study, in the form of sequenced reads, are available from the European Genome- phenome Archive (EGA) repository (https://www.ebi.ac.uk/ega/), accession numbers EGAD00001009806 (RNAseq) [80, 81] and EGAD50000000134 (ChIPseq) [82] upon request via corresponding EGA webpages. Data taken from published longitudinal bulk tumor studies is available as described in the original publications listed in Additional file 1: Table S1: EGAD00001001424 [55, 83], EGAD00001002143 [56, 84], EGAD00001004564 [2, 85], PRJNA580196 [54, 86], and from Synapse (https://www.synapse.org/glass) from which we used transcript_count_matrix_all_samples.tsv and transcript_tpm_matrix_all_samples.tsv [8, 87]. Single-cell data was acquired via EGAS00001004909 [9, 88]. Bulk tumor DNA methylation data was acquired from Synapse (https://www.synapse.org/glass) using the beta.merged.tsv file [87]. Single-cell DNA methylation data was acquired from Synapse (https://synapse.org/singlecellglioma) source file PromoterDNAmethylation.tsv [89]. ChIPseq data from primary GBM tumors was from the Sequencing Read Archive (https://www.ncbi.nlm.nih.gov/bioproject/PRJNA391756) [39, 90]. RNAseq of ex vivo GBM models was acquired from the Gene Expression Omnibus (https://www.ncbi.nlm.nih.gov/geo/query/acc.cgi?acc=GSE179649) [40, 91]. Code is available on GitHub via https://github.com/GliomaGenomics/GBM_TF_analysis and Zenodo https://doi.org/10.5281/zenodo.10423959 and is released under a Creative Commons Attribution license [92, 93].
