# Supplementary material for: IDHwt glioblastomas can be stratified by their transcriptional response to standard treatment, with implications for targeted therapy
Source: Genome Biol. 2024 Feb 7;25:45. doi: 10.1186/s13059-024-03172-3 (PMC10848526; doi:10.1186/s13059-024-03172-3)
Supplement: Supplementary file 2 — Additional file 2: Supplemental figures. This file includes 4 supplemental figures of data, along with accompanying figure legends, pertaining to this study and referenced throughout the publication. [file 13059_2024_3172_MOESM2_ESM.pptx]

## Slide 1
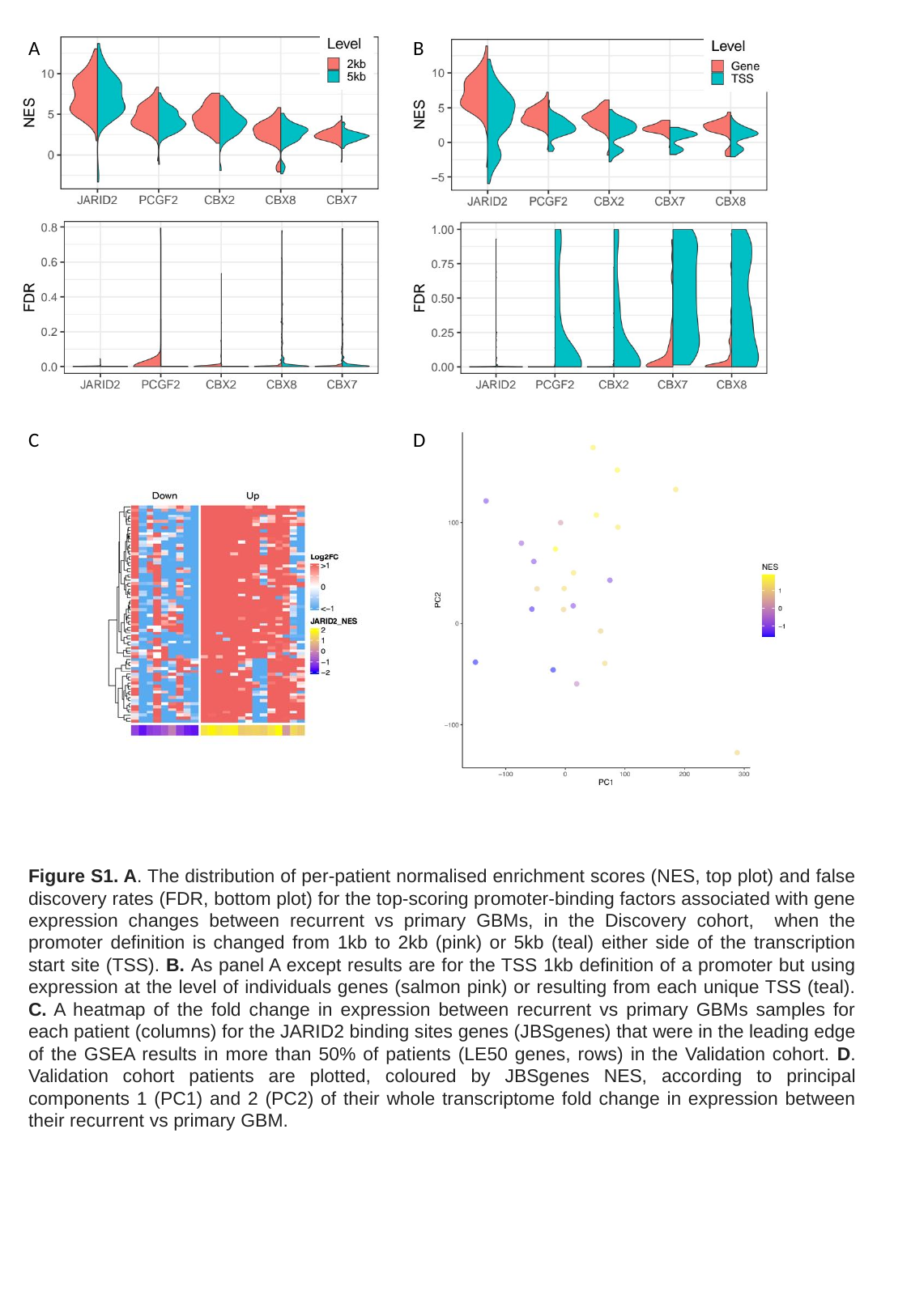

A
B
C
D

## Slide 2
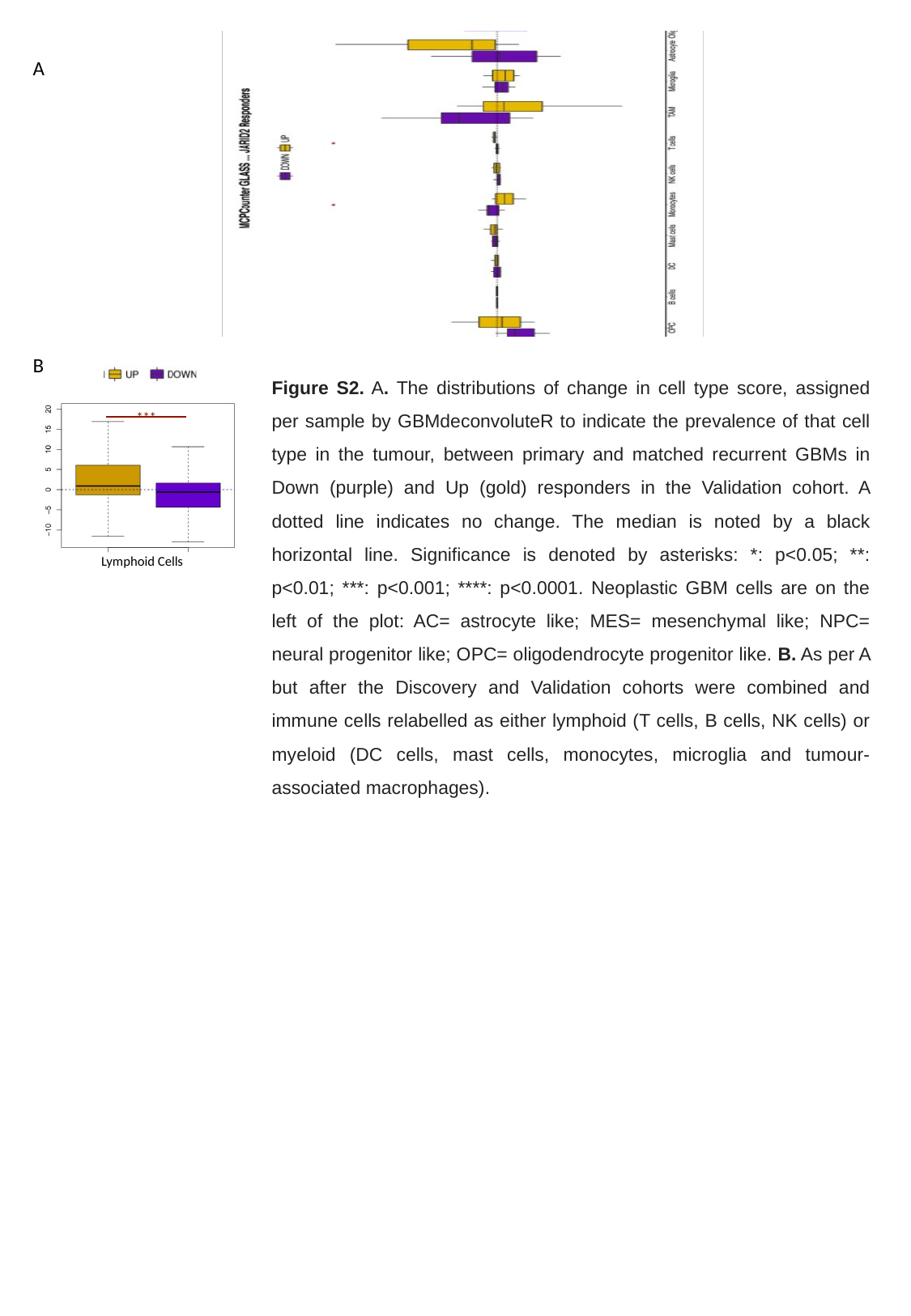

A
B
Figure S2. A. The distributions of change in cell type score, assigned per sample by GBMdeconvoluteR to indicate the prevalence of that cell type in the tumour, between primary and matched recurrent GBMs in Down (purple) and Up (gold) responders in the Validation cohort. A dotted line indicates no change. The median is noted by a black horizontal line. Significance is denoted by asterisks: *: p<0.05; **: p<0.01; ***: p<0.001; ****: p<0.0001. Neoplastic GBM cells are on the left of the plot: AC= astrocyte like; MES= mesenchymal like; NPC= neural progenitor like; OPC= oligodendrocyte progenitor like. B. As per A but after the Discovery and Validation cohorts were combined and immune cells relabelled as either lymphoid (T cells, B cells, NK cells) or myeloid (DC cells, mast cells, monocytes, microglia and tumour-associated macrophages).
***
Lymphoid Cells

## Slide 3
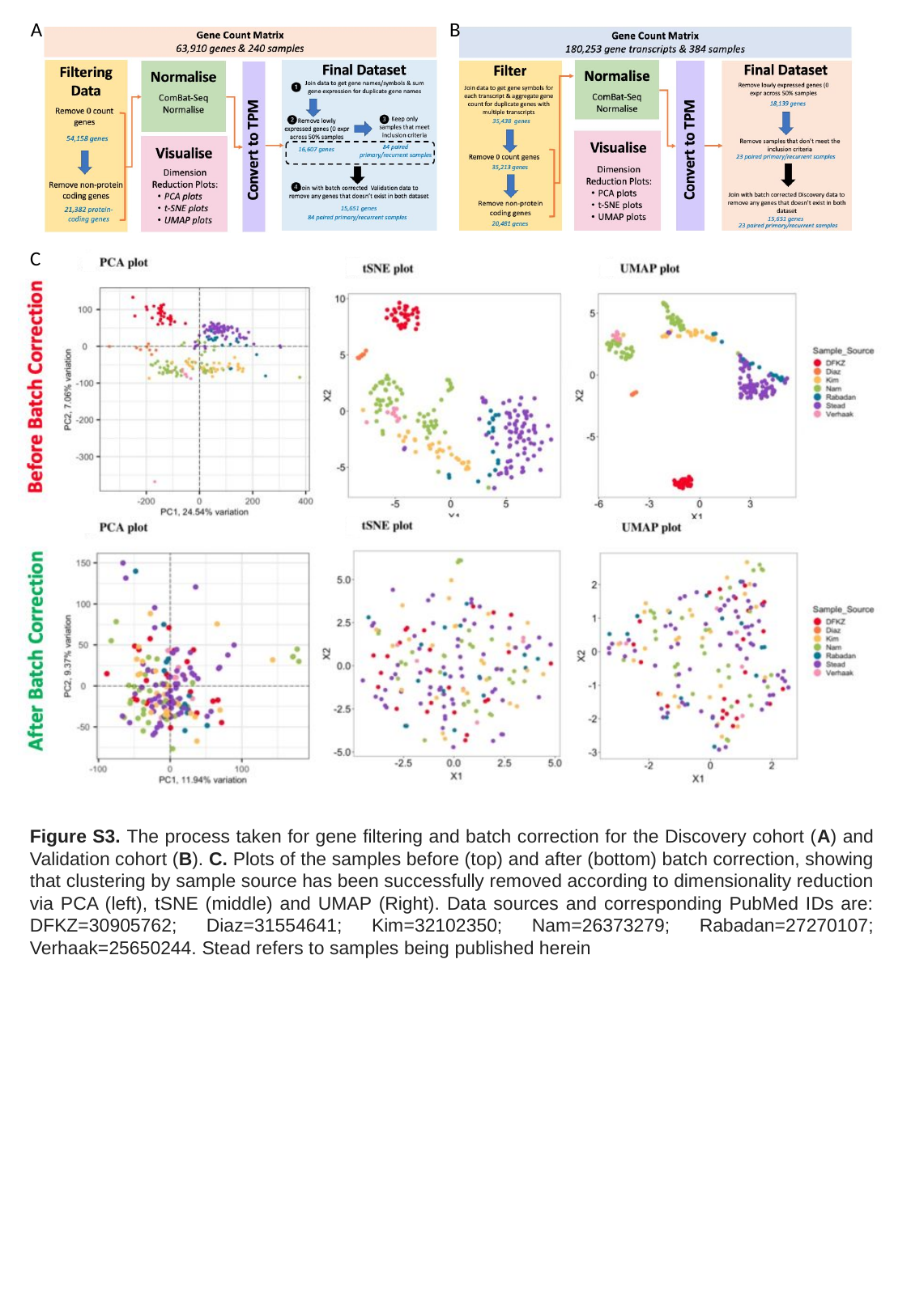

A
B
C
Figure S3. The process taken for gene filtering and batch correction for the Discovery cohort (A) and Validation cohort (B). C. Plots of the samples before (top) and after (bottom) batch correction, showing that clustering by sample source has been successfully removed according to dimensionality reduction via PCA (left), tSNE (middle) and UMAP (Right). Data sources and corresponding PubMed IDs are: DFKZ=30905762; Diaz=31554641; Kim=32102350; Nam=26373279; Rabadan=27270107; Verhaak=25650244. Stead refers to samples being published herein

## Slide 4
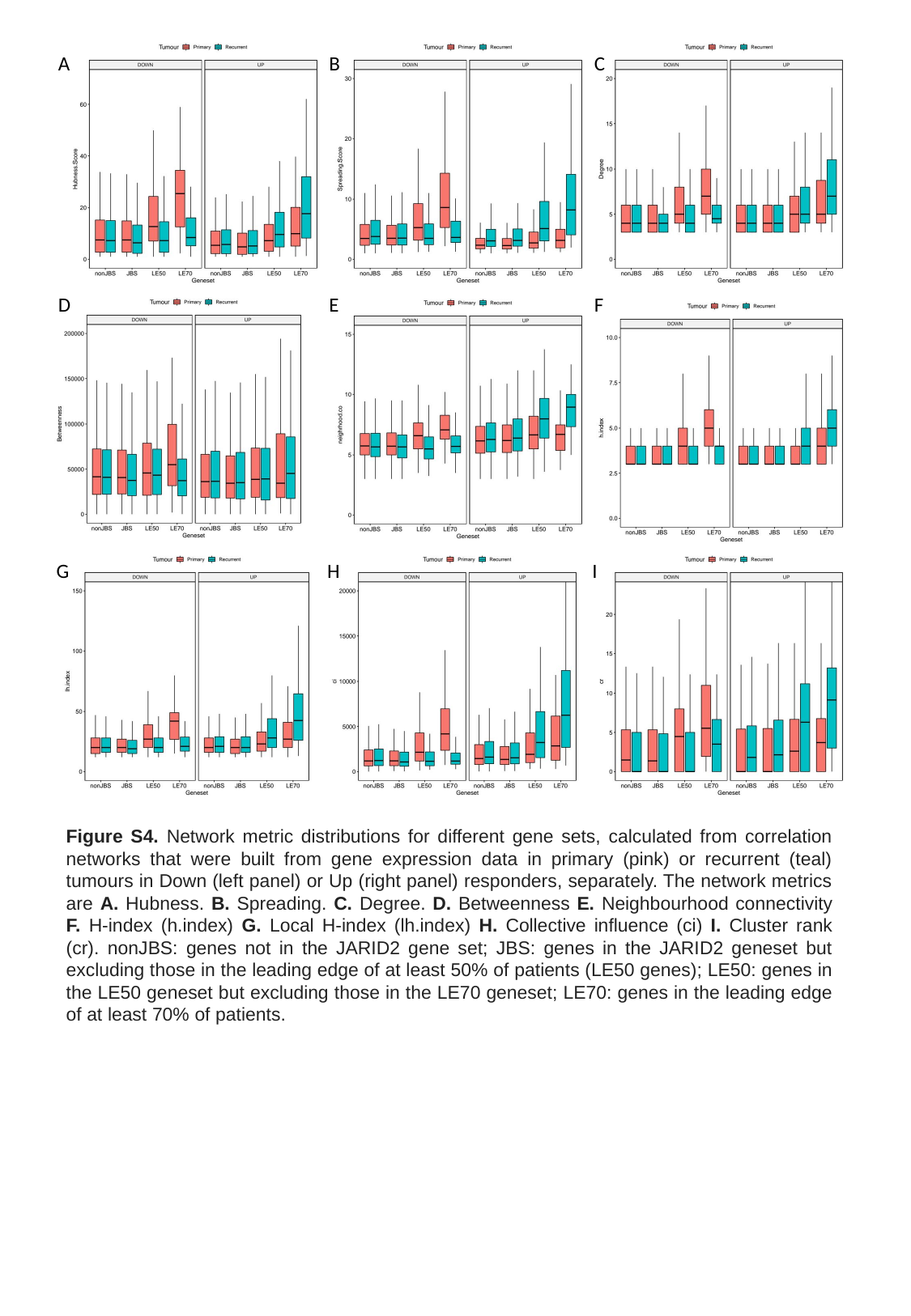

C
A
B
F
D
E
I
G
H
Figure S4. Network metric distributions for different gene sets, calculated from correlation networks that were built from gene expression data in primary (pink) or recurrent (teal) tumours in Down (left panel) or Up (right panel) responders, separately. The network metrics are A. Hubness. B. Spreading. C. Degree. D. Betweenness E. Neighbourhood connectivity F. H-index (h.index) G. Local H-index (lh.index) H. Collective influence (ci) I. Cluster rank (cr). nonJBS: genes not in the JARID2 gene set; JBS: genes in the JARID2 geneset but excluding those in the leading edge of at least 50% of patients (LE50 genes); LE50: genes in the LE50 geneset but excluding those in the LE70 geneset; LE70: genes in the leading edge of at least 70% of patients.
