# Supplementary material for: IDHwt glioblastomas can be stratified by their transcriptional response to standard treatment, with implications for targeted therapy
Source: Genome Biol. 2024 Feb 7;25:45. doi: 10.1186/s13059-024-03172-3 (PMC10848526; doi:10.1186/s13059-024-03172-3)
Supplement: Supplementary file 3 — Additional file 3. Review history. [file 13059_2024_3172_MOESM3_ESM.docx]

Review History

**First round of review**

**Reviewer 1**

**Were you able to assess all statistics in the manuscript, including the appropriateness of statistical tests used?**

Yes, and I have assessed the statistics in my report.

**Comments to author:**

Tanner et al. performed RNA-seq comparison of paired primary and recurrent GBMs (n=214), and identified a gene set potentially regulated by JARID2, whose expression is consistently up- or down-regulated from primary to recurrence, stratifying patients into Up and Down responder groups. The authors further used GABAR inhibitor treatment on cell lines belonging to Up and Down responders as an example to show these two groups behave differently in response to targeted therapy. Overall, the study expanded upon previously published bulk and single-cell analysis on primary and recurrent GBM, but some of the key conclusions and statistical analysis require further scrutiny.

Major concern:

1. Bulk RNA-seq does not distinguish whether the differentially expressed genes are contributed by tumor cells or the microenvironmental cells. Thus, the rationale for using GABAR inhibitors is not justified. Is increased GABA signaling in a subgroup of recurrent GBM due to upregulation of this pathway in tumor cells, or enrichment of microenvironmental inhibitory neuron component? The previous studies on glioma-neuron interactions show it primarily involves the excitatory, not the inhibitory synaspses. Reanalysis of the single-cell dataset (Ref. 9), along with detailed profiling of the cell lines they used, may help elucidate this point.

2. Along this line, how do the authors reconcile their conclusion with Ref. 9, which only reported a shift to a mesenchymal phenotype upon recurrence?

3. The authors try to link their DEGs to PRC2 complex. Granted, JARID2 acts as part of this complex, but is there any direct evidence to show this complex is involved? For example, are these genes downstream of Ezh2? The authors should at least show some evidence using KD or KO on cell lines.

4. The authors should look into the RNA-seq or genetic status of primary tumors, and test if there is anything in primary tumors that could predict the transition to proneural or mesenchymal status upon recurrence.

Minor issues:

1. In Fig.1B, authors performed DE analysis by DESeq2 to identify the significant genes, but a recent study (10.1186/s13059-022-02648-4) raised concern about unexpectedly high false discovery rates using DESeq2 or edgeR among human population RNA-seq samples. The authors may consider using additional statistical tools. Also, they performed enrichment analysis among genes whose FDR<0.1, why not use FDR<0.05?

2. In Fig.1G, what is "DEA"?

3. Line 307, should be Table S26 instead of S25?

**Reviewer 2**

**Were you able to assess all statistics in the manuscript, including the appropriateness of statistical tests used?**

Yes, and I have assessed the statistics in my report.

**Comments to author:**

This work is in line with several high profile manuscripts that study the longitudinal evolution of glioblastomas. The novelty presented here is the authors find two types of recurrences. Approximately 60% of recurrent samples exhibit an upregulation of genes containing a JARID2 binding site in the promoter region (Up responders), while the remaining 40% do not (Down responders). Tanner et al follow this up by a detailed investigation on the transcriptional and subtype differences between the Up and Down responders. Some of the findings, specifically the importance of GABA signalling, are corroborated through functional experiments conducted in cell culture models.

While the manuscript presents potentially intriguing findings, it fails to support the conclusions drawn by the authors. Firstly, it is important to note that some patients underwent surgery with very short intervals (table S1). Such short intervals are often employed to remove residual tissue rather than indicating tumor progression. Therefore, these samples should be considered to be derived from the same tumor. Second, glioblastomas are invasive tumors and therefore are a mixture of various cell types. Several reports have now found that tumor purity changes as it progresses and this confounding factor may drastically affect conclusions. Lastly, throughout the manuscript, the authors assume that the observed changes are attributable to treatment. This conclusion cannot be drawn as it was not a covariate in their analysis.

In addition to these points, I believe that the functional experiments conducted in this study are insufficient to fully support the presented findings. The experiments were performed using only two cell cultures of which one is a standard cell line, unlikely to reflect the GBM in situ. Addition of GABA (ant)agonists provides a hint to confirm the authors conclusion, but drugs have strong side effects too. knockout or downregulation of the target genes could provide a more precise and controlled model system. There is no analysis of subtype switching in these models. The addition of JARID2 reporter constructs could also help, as would immunofluorescent stainings for the candidate genes.

These additional approaches would enhance the comprehensiveness and validity of the study's findings.

Other points:

There are more samples in the GLASS cohort for validation and it is not described why only a fraction of the GLASS database was leveraged.

The discovery cohort seems a collection of samples from different sources and different sequencing batches. Although for statistics in a fully paired manner (as applied here) further batch corrections are unnecessary, for visualizations such as resection level clustering this needs to be taken into consideration. Batches that require correction in this context should be provided and detailed in the methods.

The statement "we created novel, comprehensive gene sets for DNA-binding factors" requires a deeper explanation in results section to guide the reader

It would also be of interest to explore correlation of Up and Down responders with patient outcome (even if there is none), and with genetic changes (I suspect the genetic changes of the GLASS samples used in this study are known).

**Reviewer 3**

**Were you able to assess all statistics in the manuscript, including the appropriateness of statistical tests used?**

No, I do not feel adequately qualified to assess the statistics.

**Comments to author:**

The manuscript by Tanner et al describes RNAseq data from 107 pairs of pre and post treatment IDH WT human glioblastomas (GBM) specimens. The primary finding is that a group of genes containing JARID2 promoter binding sites change their expression after treatment, with about 2/3 showing upregulation and 1/3 downregulation. Several comments:

1) The dataset is large, unique and valuable. The observations are very interesting and could be translated clinically. Interestingly, they identify two cell lines with the same up or down phenotypes after treatment.

2) A shortcoming is that the study primarily involves RNAseq data. The investigators have found a limited number of genes with opposite transcriptional responses, and have indicated that they cannot predict before treatment whether there will be up or down regulation. Missing are data relevant on whether the promoters of these genes are epigenetically different. The authors hint at "epigenetic remodeling complex" (bottom of page 2), and potentially the promoters have different epigenetic states prior to treatment. This important issue is not well-addressed, but could easily be addressed with public data (methylation or ChIP-Seq) showing whether or not IDH WT GBMs have heterogenous epigenetic configurations at these genes. (Ideally these studies could be performed on up or down tumors, perhaps on the two up or down cell lines in this paper). Even negative results (ie no difference in promoter epigenetics) would be valuable because different epigenetic promotor configurations seems like a logical mechanism for the opposite transcriptional responses.

Minor points

1) It's a bit confusing to call relapses as "responders" albeit the response indicates a transcriptional change (maybe "Up and Down Relapses").

2) page 5 is very dense (maybe breakup into paragraphs).

**Authors Response**

**Point-by-point responses to the reviewers’ comments:**

Reviewer #1: Tanner et al. performed RNA-seq comparison of paired primary and recurrent GBMs (n=214), and identified a gene set potentially regulated by JARID2, whose expression is consistently up- or down-regulated from primary to recurrence, stratifying patients into Up and Down responder groups. The authors further used GABAR inhibitor treatment on cell lines belonging to Up and Down responders as an example to show these two groups behave differently in response to targeted therapy. Overall, the study expanded upon previously published bulk and single-cell analysis on primary and recurrent GBM, but some of the key conclusions and statistical analysis require further scrutiny. 

Major concern:
1. Bulk RNA-seq does not distinguish whether the differentially expressed genes are contributed by tumor cells or the microenvironmental cells. Thus, the rationale for using GABAR inhibitors is not justified. Is increased GABA signaling in a subgroup of recurrent GBM due to upregulation of this pathway in tumor cells, or enrichment of microenvironmental inhibitory neuron component? The previous studies on glioma-neuron interactions show it primarily involves the excitatory, not the inhibitory synapses. Reanalysis of the single-cell dataset (Ref. 9), along with detailed profiling of the cell lines they used, may help elucidate this point.

*Reply: Thank you for the comment but also the suggestions on how to address the TME issue in particular. We also felt this needed more exploration and have done the below to probe further.*

- *We have now incorporated normal brain cell marker genes into our bulk RNAseq deconvolution tool and were able to show that longitudinal shifts in normal brain cell populations are distinct between responder subtypes i.e. normal brain cells are upregulated uniquely in Up responders, further suggesting that glioma:neuronal interactions are important in this specific subset of patients. The results section was renamed “Responder subtypes undergo different changes in neoplastic,* ***normal brain*** *and immune cell populations through treatment” and amended (lines 373-443) and new plots were made - now* ***Fig 2C*** *and* ***S2A***
- *We downloaded longitudinal single cell data from 22 patients from Ref 9 and analysed each dataset with neoplastic, immune and normal brain cells separated out. This confirms that the genes we see changing expression over time are indeed dysregulated in the GBM cancer cells. However, results also show that the dysregulation is also present, but opposite, in the surrounding normal brain, suggesting that interaction with the TME may be associated with this phenomenon via potential feedback loops. We created an entirely new results section (lines 445-486)* ***“GBM responder subtyping is driven by neoplastic cells but impacted by tumour purity”****, complete with novel accompanying figure (now* ***Fig 3****)*
- *We characterised additional cell lines to acquire one Up and one Down responder per established cell lines and patient derived cell lines (known to represent different tumour biology in GBM), and further showed that adding serum to patient derived cell lines did not alter the responder subtype. We include data showing how the responder subtype is evident in these cell lines meaning, alongside the above, that this is a cancer-cell intrinsic property. However, the effect size in pure cell lines is much smaller than that seen in patients. Based on the additional analyses we outline above, we hypothesised that the lower effect size was owing to a lack of TME in our model system. To probe this further we acquired data from organotypic GBM slices that were cultured and treated with chemoradiation ex vivo prior to RNAseq. We use these data to show that the act of culturing did not lower the effect size, which was more aligned with patients in these TME-containing models. Such models are not amenable to target validation and functional genomics, so we conclude that additional investigation of model systems is required. We have altered the title of the pertinent results section to “Responder subtypes are recapitulated in preclinical models of GBM* ***but tumour microenvironment is required****” and added the info on the additional cell lines. We also added new paragraphs on the ex vivo data analyses (lines 723 to 731) and made significant changes to what is now* ***Fig. 7.*** *We have also added this to the discussion (lines 1059-1086).*
- *We agree that inhibitor studies are a rather blunt instrument here and believe that functional genomics approaches would be more suitable for assessing the effect of candidate targets for each responder subtype. However, as per the preceding bullet point, we need to identify a more suitable model system that incorporates the TME (such as co-culture with cortical organoids) that can be used screening approaches. We have, thus, removed the inhibitor experiments from our paper and suggested that model optimisation is first required. In strong support of this, since we submitted a new study has linked gene expression in GBM to significant redistribution of histone marks in regulatory regions (including promoters) in ways which particularly alter neuronal signalling and interaction [Chakraborty et al. Rewiring of the promoter-enhancer interactome and regulatory landscape in glioblastoma orchestrates gene expression underlying neurogliomal synaptic communication. Nat Commun 2023, 14:6446]. This study specifically shows that the changes to the observed rewiring (in comparison to normal brain) by pharmacological manipulation is only evident in GBM cell lines when they are co-cultured with neurons. Hence, this further shows that the right model system must be identified prior to any downstream validation of our work. The study, and its relevance to our work, have been added to the discussion (lines 1059-1086).*

2. Along this line, how do the authors reconcile their conclusion with Ref. 9, which only reported a shift to a mesenchymal phenotype upon recurrence?

*Reply: This is a great point and, in fact our senior author, Dr Stead, wrote a review of this study for Nature Cancer, in which she highlighted that their data does not support a shift in a single direction, but rather shows that there is a bi-directional shift (see below Fig 3a taken from Ref 9 where going from P to R there are some lines increasing but others are clearly reducing). We have explicitly included this point in the discussion (lines 933-937).*

3. The authors try to link their DEGs to PRC2 complex. Granted, JARID2 acts as part of this complex, but is there any direct evidence to show this complex is involved? For example, are these genes downstream of Ezh2? The authors should at least show some evidence using KD or KO on cell lines.

*Reply: Thank you for this suggestion. Based on the response to point 1 (final bullet point), we need to identify a suitable model in which to perform functional genomics studies, including to ascertain the epigenetic mechanisms upstream of gene dysregulation. However, we have now performed several new analyses and acquired novel ChIPseq data that supports the involvement of PRC2, via its unique histone modification: H3K27me3. This has been included as an entirely new results section (lines 629-682) “****JBSgenes are hypomethylated and significantly associated with the PRC2 histone mark: H3K27me3****” and led to a completely new figure (now* ***Fig. 6****). To outline specifically what these new data are:*

1. *Our new single cell analyses highlights EZH2 as the second most enriched DNA binding factor in cancer cells specifically (now* ***Fig 3A****)*
2. *We acquired and analysed bulk longitudinal and single cell DNA methylation data to provide evidence that the promoter DNA at the genes in question (our JBSgenes) is significantly hypomethylated, in Up and Down responders in both primary and recurrent samples; and that these genes are seldomly differentially methylated longitudinally. This suggests that the gene dysregulation is NOT caused by DNA methylation. This is included in results (lines 631 to 650 and as* ***Figs. 6A-D****)*
3. *We have acquired H3K27me3 profiling data from 9 primary patient GBMs (*[*https://pubmed.ncbi.nlm.nih.gov/29549165/*](https://pubmed.ncbi.nlm.nih.gov/29549165/)*) and shown that JBSgenes are significantly associated with this PRC2-specific histone mark (lines 652 to 667 and, now,* ***Fig. 6E****)*
4. *We have performed ChIPseq to profile EZH2 binding in two longitudinal pairs of tumours from our cohort: one Up and one Down responder. This clearly shows a significant association of EZH2 at the JBSgenes in GBM, and distinct changes in occupancy of EZH2 over time between the responder subtypes in ways that support the observed gene expression changes (lines 667-682 and, now,* ***Figs. 6F-G****).*

4. The authors should look into the RNA-seq or genetic status of primary tumors, and test if there is anything in primary tumors that could predict the transition to proneural or mesenchymal status upon recurrence.

*Reply: We are very much in agreement that we need to look at prediction capability. However, although there is a preferential shift to proneural or mesenchymal in our responder subtypes it is not an exclusive phenomenon so we are trying to predict responder subtype at initial diagnosis. Our initial investigation has indicated that responder subtype cannot be predicted by primary tumour gene expression alone, though this might be owing to the cohort size (which, whilst considerable with regards molecular profiling studies, is small for machine learning based classification research) -* [*https://academic.oup.com/neuro-oncology/article/25/Supplement_3/iii13/7275511*](https://academic.oup.com/neuro-oncology/article/25/Supplement_3/iii13/7275511)*. We now have a PhD project running 2023-2026 which is using AI tools to see if we can predict responder subtype by integrating several data types (expression, mutations, copy number, imaging and metabolomics). We have chosen not to include our preliminary negative results in this manuscript, in favour of completing this work more robustly and publishing it then.*

Minor issues:
1. In Fig.1B, authors performed DE analysis by DESeq2 to identify the significant genes, but a recent study (10.1186/s13059-022-02648-4) raised concern about unexpectedly high false discovery rates using DESeq2 or edgeR among human population RNA-seq samples.

*Reply: Thank you for alerting us to this issue, which we considered and performed some extra analysis to address, as described here. DEseq2 is a widely used and accepted tool. We have not, per se, relied on a single differentially expressed gene result but, rather, investigated pathways of genes (which would be harder to identify in noisy data with lots of false positives). However, to determine if using a different tool would alter our findings, we re-ran our initial primary versus recurrent differential expression analysis using the Wilcoxon rank-sum method as recommended by the Li et al. authors, following their guide (*[*https://rpubs.com/LiYumei/806213*](https://eur03.safelinks.protection.outlook.com/?url=https%3A%2F%2Frpubs.com%2FLiYumei%2F806213&data=05%7C01%7CL.F.Stead%40leeds.ac.uk%7Cbf5c256c822546aa82d908db68151122%7Cbdeaeda8c81d45ce863e5232a535b7cb%7C1%7C0%7C638218211950655208%7CUnknown%7CTWFpbGZsb3d8eyJWIjoiMC4wLjAwMDAiLCJQIjoiV2luMzIiLCJBTiI6Ik1haWwiLCJXVCI6Mn0%3D%7C3000%7C%7C%7C&sdata=qmFeMgmov1AhnvDYhz8P159kzqW2jO0zx6NHeeBO0rw%3D&reserved=0)*), adapted for a paired analysis. We had a 56% overlap in the most highly ranked, by significance, genes and a highly significant overlap (p<1E-16) in the resulting pathways that were enriched (which constituted the basis for our findings) by both methods. We, thus, feel confident in retaining our results from DESeq2 analyses.*

The authors may consider using additional statistical tools. Also, they performed enrichment analysis among genes whose FDR<0.1, why not use FDR<0.05?

*Reply: Apologies for reporting that we used FDR<0.1: this was a typo and we were actually more stringent and used FDR<0.01 as now corrected on line 835.*

2. In Fig.1G, what is "DEA"?

*Reply: Sorry for the oversight. DEA = Differential Expression Analysis. This has now been added in the figure legend.*

3. Line 307, should be Table S26 instead of S25?

*Reply: Our apologies: we have changed this (now line 509).*

Reviewer #2: This work is in line with several high profile manuscripts that study the longitudinal evolution of glioblastomas. The novelty presented here is the authors find two types of recurrences. Approximately 60% of recurrent samples exhibit an upregulation of genes containing a JARID2 binding site in the promoter region (Up responders), while the remaining 40% do not (Down responders). Tanner et al follow this up by a detailed investigation on the transcriptional and subtype differences between the Up and Down responders. Some of the findings, specifically the importance of GABA signalling, are corroborated through functional experiments conducted in cell culture models.

While the manuscript presents potentially intriguing findings, it fails to support the conclusions drawn by the authors. Firstly, it is important to note that some patients underwent surgery with very short intervals (table S1). Such short intervals are often employed to remove residual tissue rather than indicating tumor progression. Therefore, these samples should be considered to be derived from the same tumor.

*Reply: This is a pertinent point, thank you for raising it. It has now been highlighted in the discussion in lines (905-919). In summary: herein, we consider surgery + radiation + temozolomide as the treatment. Any viable tumour after those treatments have been received is of interest to us as it has ‘evaded’ removal/killing by multi-modal treatment and, thus, constitutes post-treatment surviving tumour. Hence, we now explicitly state that recurrences following shorter surgical intervals are unlikely to be tumour regrowth rather than survival of a persistent tumour.*

Second, glioblastomas are invasive tumors and therefore are a mixture of various cell types. Several reports have now found that tumor purity changes as it progresses and this confounding factor may drastically affect conclusions.

*Reply: We were also concerned about this potential confounder so thank you to this referee for further highlighting it. We have managed to do quite a lot of extra work which gives us great confidence that we are looking at a cancer intrinsic phenomenon, but one in which tumour purity must be taken into account as explained here:*

- *We have now incorporated normal brain cell marker genes into our bulk RNAseq deconvolution tool and were able to show that longitudinal shifts in normal brain cell populations are distinct between responder subtypes i.e. normal brain cells are upregulated uniquely in Up responders, further suggesting that glioma:neuronal interactions are important in this specific subset of patients. The results section was renamed “Responder subtypes undergo different changes in neoplastic,* ***normal brain*** *and immune cell populations through treatment” and amended (lines 373-443) and new plots were made - now* ***Fig 2C*** *and* ***S2A.***
- *We downloaded longitudinal single cell data from 22 patients from Ref 9 and analysed each dataset with neoplastic, immune and normal brain cells separated out. This confirms that the genes we see changing expression over time are indeed dysregulated in the GBM cancer cells. However, results also show that the dysregulation is also present, but opposite, in the surrounding normal brain, suggesting that interaction with the TME may be associated with this phenomenon via potential feedback loops. We created an entirely new results section (lines 445-486)* ***“GBM responder subtyping is driven by neoplastic cells but impacted by tumour purity”****, complete with novel accompanying figure (now* ***Fig 3****).*

Lastly, throughout the manuscript, the authors assume that the observed changes are attributable to treatment. This conclusion cannot be drawn as it was not a covariate in their analysis.

*Reply: This is a valid and important point. We have carefully gone through the manuscript and reworded to try and remove any indication that we can conclude that this is a treatment driven phenomenon. We have also explicitly added this point to the discussion (lines 905-919), alongside noting the difficulty in acquiring similar numbers of paired samples that did not receive treatment (and actually impossible to acquire these if we consider that surgery is part of treatment). We go on to say that this further highlights the need to identify models that adequately recapitulate the phenomenon for testing and screening in vitro. This is in lines 988-990 (on which we have done extra work, as detailed in response to the comment below).*

In addition to these points, I believe that the functional experiments conducted in this study are insufficient to fully support the presented findings. The experiments were performed using only two cell cultures of which one is a standard cell line, unlikely to reflect the GBM in situ. Addition of GABA (ant)agonists provides a hint to confirm the authors conclusion, but drugs have strong side effects too. knockout or downregulation of the target genes could provide a more precise and controlled model system.

*Reply: To address this valid point we have done extra work as outlined below:*

*We characterised additional cell lines to acquire one Up and one Down responder per established cell lines and patient derived cell lines (known to represent different tumour biology in GBM), and further showed that adding serum to patient derived cell lines did not alter the responder subtype. We include data showing how the responder subtype is evident in these cell lines meaning, alongside the above, that this is a cancer-cell intrinsic property. However, the effect size in pure cell lines is much smaller than that seen in patients. Based on the additional analyses we outline above; we hypothesised that the lower effect size was owing to a lack of TME in our model system. To probe this further we acquired data from organotypic GBM slices that were cultured and treated with chemoradiation ex vivo prior to RNAseq. We use these data to show that the act of culturing did not lower the effect size, which was more aligned with patients in these TME-containing models. Such models are not amenable to target validation and functional genomics, so we conclude that additional investigation of model systems is required. We have altered the title of the pertinent results section to “Responder subtypes are recapitulated in preclinical models of GBM* ***but tumour microenvironment is required****” and added the info on the additional cell lines. We also added new paragraphs on the ex vivo data analyses (lines 723 to 731) and made significant changes to what is now* ***Fig. 7.*** *We have also added this to the discussion (lines 1059-1086).*

*We agree that inhibitor studies are a rather blunt instrument here and believe that functional genomics approaches would be more suitable for assessing the effect of candidate targets for each responder subtype. However, as per the preceding bullet point, we need to identify a more suitable model system that incorporates the TME (such as co-culture with cortical organoids) that can be used screening approaches. We have, thus, removed the inhibitor experiments from our paper and suggested that model optimisation is first required. In strong support of this, since we submitted a new study has linked gene expression in GBM to significant redistribution of histone marks in regulatory regions (including promoters) in ways which particularly alter neuronal signalling and interaction [Chakraborty et al. Rewiring of the promoter-enhancer interactome and regulatory landscape in glioblastoma orchestrates gene expression underlying neurogliomal synaptic communication. Nat Commun 2023, 14:6446]. This study specifically shows that the changes to the observed rewiring (in comparison to normal brain) by pharmacological manipulation is only evident in GBM cell lines when they are co-cultured with neurons. Hence, this further shows that the right model system must be identified prior to any downstream validation of our work. The study, and its relevance to our work, have been added to the discussion (lines 1059-1086).*

There is no analysis of subtype switching in these models. The addition of JARID2 reporter constructs could also help, as would  immunofluorescent stainings for the candidate genes. These additional approaches would enhance the comprehensiveness and validity of the study's findings.

*Reply: As per the response to the above comment, we now realise that we need to identify a suitable model in which to perform further preclinical/experimental data analyses and functional genomics studies. This will very much be the focus of our future work to be published in follow on papers.*

Other points:
There are more samples in the GLASS cohort for validation and it is not described why only a fraction of the GLASS database was leveraged.

*Reply: We have added to the results (lines 234-236) the explanation that a) some of the GLASS samples are included in our Discovery cohort because we had access to the raw data, and b) GLASS is a superb resource but there is a lack of clinical metadata with many samples which negated their inclusion due to our strict inclusion criteria (GBM IDHwt at both time points, local recurrence having undergone standard treatment).*

*However, the single cell Diaz data that we are now including is a further 22 patients (44 samples).*

The discovery cohort seems a collection of samples from different sources and different sequencing batches. Although for statistics in a fully paired manner (as applied here) further batch corrections are unnecessary, for visualizations such as resection level clustering this needs to be taken into consideration. Batches that require correction in this context should be provided and detailed in the methods.

*Reply: This is very true. We have performed significant batch correction and have included details in the methods (new section ‘****Batch Correction’*** *on lines 1185 to 1192) with accompanying new supplemental figure (****Fig. S3****) and alluded to it in the results (line 569).*

The statement "we created novel, comprehensive gene sets for DNA-binding factors" requires a deeper explanation in results section to guide the reader.

*Reply: Thank you for point this out. Extra detail has been added on lines 327-330.*

It would also be of interest to explore correlation of Up and Down responders with patient outcome (even if there is none), and with genetic changes (I suspect the genetic changes of the GLASS samples used in this study are known).

*Reply: The exploration of association with patient outcome is in lines 376-379.*

*Unfortunately, very few of the samples from GLASS also happen to be the ones that include genetic data. We did perform variant calling on the RNAseq data but no clear genetic differences were apparent. We now have a PhD project running 2023-2026 which is using AI tools to see if we can predict responder subtype at primary diagnosis by integrating several data types (expression, mutations, copy number, imaging and metabolomics). We have chosen not to include our preliminary negative results in this manuscript, in favour of completing this work more robustly and publishing it then.*

Reviewer #3: The manuscript by Tanner et al describes RNAseq data from 107 pairs of pre and post treatment IDH WT human glioblastomas (GBM) specimens. The primary finding is that a group of genes containing JARID2 promoter binding sites change their expression after treatment, with about 2/3 showing upregulation and 1/3 downregulation.

Several comments:
1) The dataset is large, unique and valuable. The observations are very interesting and could be translated clinically. Interestingly, they identify two cell lines with the same up or down phenotypes after treatment.

*Reply: Many thanks for your positive comments and reflection on the interest and potential applicability of our work.*

2) A shortcoming is that the study primarily involves RNAseq data. The investigators have found a limited number of genes with opposite transcriptional responses, and have indicated that they cannot predict before treatment whether there will be up or down regulation. Missing are data relevant on whether the promoters of these genes are epigenetically different. The authors hint at "epigenetic remodeling complex" (bottom of page 2), and potentially the promoters have different epigenetic states prior to treatment. This important issue is not well-addressed, but could easily be addressed with public data (methylation or ChIP-Seq) showing whether or not IDH WT GBMs have heterogenous epigenetic configurations at these genes. (Ideally these studies could be performed on up or down tumors, perhaps on the two up or down cell lines in this paper). Even negative results (i.e., no difference in promoter epigenetics) would be valuable because different epigenetic promotor configurations seems like a logical mechanism for the opposite transcriptional responses.

*Reply: Thank you for pointing out the strength in further exploring the epigenetic aspect which we have done as outlined here:*

- *We have now performed several new analyses and acquired novel ChIPseq data that supports the involvement of PRC2, via its unique histone modification: H3K27me3. This has been included as an entirely new results section (lines 629-682) “****JBSgenes are hypomethylated and significantly associated with the PRC2 histone mark: H3K27me3****” and led to a completely new figure (now* ***Fig. 6****). To outline specifically what these new data are:*
  - *We performed new single cell RNAseq data analyses, which highlights EZH2 as the second most enriched DNA binding factor in cancer cells specifically (now* ***Fig 3A****)*
  - *We acquired and analysed bulk longitudinal and single cell DNA methylation data to provide evidence that the promoter DNA at the genes in question (our JBSgenes) is significantly hypomethylated, in Up and Down responders in both primary and recurrent* *samples; and that these genes are seldomly differentially methylated longitudinally. This suggests that the gene dysregulation is NOT caused by DNA methylation. This is included in results (lines 631 to 650 and as* ***Figs. 6A-D****).*
  - *We have acquired H3K27me3 profiling data from 9 primary patient GBMs (*[*https://pubmed.ncbi.nlm.nih.gov/29549165/*](https://pubmed.ncbi.nlm.nih.gov/29549165/)*) and shown that JBSgenes are significantly associated with this PRC2-specific histone mark (lines 652 to 667 and, now,* ***Fig. 6E****).*
  - *We have performed ChIPseq to profile EZH2 binding in two longitudinal pairs of tumours from our cohort: one Up and one Down responder. This clearly shows a significant association of EZH2 at the JBSgenes in GBM, and distinct changes in occupancy of EZH2 over time between the responder subtypes in ways that support the observed gene expression changes (lines 667-682 and, now,* ***Figs. 6F-G****).*
- *We have not performed additional epigenetic profiling of our cell lines that we have as these need to be further optimised owing to the following new analyses. We characterised additional cell lines to acquire one Up and one Down responder per established cell lines and patient derived cell lines (known to represent different tumour biology in GBM), and further showed that adding serum to patient derived cell lines did not alter the responder subtype. We include data showing how the responder subtype is evident in these cell lines meaning, alongside the above, that this is a cancer-cell intrinsic property. However, the effect size in pure cell lines is much smaller than that seen in patients. Based on the additional analyses we outline above, we hypothesised that the lower effect size was owing to a lack of TME in our model system. To probe this further we acquired data from organotypic GBM slices that were cultured and treated with chemoradiation ex vivo prior to RNAseq. We use these data to show that the act of culturing did not lower the effect size, which was more aligned with patients in these TME-containing models. Such models are not amenable to target validation and functional genomics, so we conclude that additional investigation of model systems is required. We have altered the title of the pertinent results section to “Responder subtypes are recapitulated in preclinical models of GBM* ***but tumour microenvironment is required****” and added the info on the additional cell lines. We also added new paragraphs on the ex vivo data analyses (lines 723 to 731) and made significant changes to what is now* ***Fig. 7.*** *We have also added this to the discussion (lines 1059-1086).*

Minor points
1) It's a bit confusing to call relapses as "responders" albeit the response indicates a transcriptional change (maybe "Up and Down Relapses")

*Reply: We really pondered on this and subsequently discussed it with neuro-oncology colleagues at length . We, respectfully, wish to keep with the terminology we opted for because patients are only stratified (at present) once they know their (transcriptional) response over time. The issue with calling these Up and Down Relapses is that the recurrent tumour is commonly referred to as the relapse whereas we want to use terminology that references the patient, not the tumour at either time point.*

2) page 5 is very dense (maybe breakup into paragraphs)

*Reply: We agree and have broken the text up as suggested.*

**Second round of review**

**Reviewer 1**

The authors have properly addressed my concerns. I have no more comments.

**Reviewer 2**

The authors have put in considerable effort in this revised manuscript. They have addressed my points of improvement and I have no further comments.

**Reviewer 3**

The authors have responded to my concerns by more fully characterizing the epigenetic differences between their two types of GBM responses.
